# The Two-Component System RsrS-RsrR Regulates the Tetrathionate Intermediate Pathway for Thiosulfate Oxidation in *Acidithiobacillus caldus*

**DOI:** 10.3389/fmicb.2016.01755

**Published:** 2016-11-03

**Authors:** Zhao-Bao Wang, Ya-Qing Li, Jian-Qun Lin, Xin Pang, Xiang-Mei Liu, Bing-Qiang Liu, Rui Wang, Cheng-Jia Zhang, Yan Wu, Jian-Qiang Lin, Lin-Xu Chen

**Affiliations:** ^1^State Key Laboratory of Microbial Technology, Shandong UniversityJinan, China; ^2^School of Mathematics, Shandong UniversityJinan, China

**Keywords:** RsrS-RsrR, two-component system, *Acidithiobacillus caldus*, sulfur metabolism, thiosulfate oxidation, S_4_I pathway, transcriptional regulation, *cis* regulatory element

## Abstract

*Acidithiobacillus caldus* (*A. caldus*) is a common bioleaching bacterium that possesses a sophisticated and highly efficient inorganic sulfur compound metabolism network. Thiosulfate, a central intermediate in the sulfur metabolism network of *A. caldus* and other sulfur-oxidizing microorganisms, can be metabolized via the tetrathionate intermediate (S_4_I) pathway catalyzed by thiosulfate:quinol oxidoreductase (Tqo or DoxDA) and tetrathionate hydrolase (TetH). In *A. caldus*, there is an additional two-component system called RsrS-RsrR. Since *rsrS* and *rsrR* are arranged as an operon with *doxDA* and *tetH* in the genome, we suggest that the regulation of the S_4_I pathway may occur via the RsrS-RsrR system. To examine the regulatory role of the two-component system RsrS-RsrR on the S_4_I pathway, Δ*rsrR* and Δ*rsrS* strains were constructed in *A. caldus* using a newly developed markerless gene knockout method. Transcriptional analysis of the *tetH* cluster in the wild type and mutant strains revealed positive regulation of the S_4_I pathway by the RsrS-RsrR system. A 19 bp inverted repeat sequence (IRS, AACACCTGTTACACCTGTT) located upstream of the *tetH* promoter was identified as the binding site for RsrR by using electrophoretic mobility shift assays (EMSAs) *in vitro* and promoter-probe vectors *in vivo*. In addition, Δ*rsrR*, and Δ*rsrS* strains cultivated in K_2_S_4_O_6_-medium exhibited significant growth differences when compared with the wild type. Transcriptional analysis indicated that the absence of *rsrS* or *rsrR* had different effects on the expression of genes involved in sulfur metabolism and signaling systems. Finally, a model of tetrathionate sensing by RsrS, signal transduction via RsrR, and transcriptional activation of *tetH*-*doxDA* was proposed to provide insights toward the understanding of sulfur metabolism in *A. caldus*. This study also provided a powerful genetic tool for studies in *A. caldus*.

## Introduction

Sulfur oxidizing microorganisms, widely distributed within the chemoautotrophic bacteria and archaea (Goebel and Stackebrandt, 1994; Friedrich, 1997; Suzuki, 1999; Kletzin et al., 2004; Friedrich et al., 2005; Frigaard and Dahl, 2008; Ghosh and Dam, 2009), have evolved a variety of sulfur redox enzymes to metabolize elemental sulfur and various reduced inorganic sulfur compounds (RISCs). Thiosulfate, a central intermediate, plays a key role in inorganic sulfur metabolism in these sulfur oxidizers (Friedrich et al., 2005; Ghosh and Dam, 2009). It is metabolized mainly through the sulfur oxidizing (Sox) enzyme system and the tetrathionate intermediate (S_4_I) pathway. The Sox system, composed of SoxYZ, SoxAX, SoxB, and Sox(CD)_2_ (Friedrich et al., 2000, 2005), completely decomposes thiosulfate to sulfate without generating any sulfur intermediates. Many acidophiles (Friedrich et al., 2005; Ghosh and Dam, 2009; Williams and Kelly, 2013) have a truncated Sox system without Sox(CD)_2_ (Dahl and Prange, 2006). The alternate S_4_I pathway is widely found in chemoautotrophic genera including *Acidithiobacillus, Thermithiobacillus, Halothiobacillus*, and *Tetrathiobacter* (Dam et al., 2007; Ghosh and Dam, 2009). This pathway is made up of a thiosulfate:quinol oxidoreductase (Tqo or DoxDA) and a tetrathionate hydrolase (TetH). DoxDA oxidizes thiosulfate to tetrathionate, while TetH hydrolyzes tetrathionate to thiosulfate and other products (Hallberg et al., 1996; Ghosh and Dam, 2009). Thus, the Sox and S_4_I pathways play important roles in the metabolism of RISCs in sulfur-oxidizing microorganisms.

*Acidithiobacillus caldus* (*A. caldus*) is an obligate chemoautotrophic sulfur-oxidizing bacterium and one of the most abundant microorganisms in industrial bioleaching systems (Hallberg and Lindström, 1994, 1996; Rawlings, 1998; Dopson and Lindström, 1999). *A. caldus* possesses a truncated Sox system encoded by two *sox* clusters (*sox*-I and *sox*-II) and also has a typical S_4_I pathway encoded by a *tetH* cluster (Valdés et al., 2008, 2009; Chen et al., 2012). Furthermore, sulfur metabolism also occurs by other enzymes in this organism. A sulfur quinone oxidoreductase enzyme (SQR) is responsible for oxidation of hydrogen sulfide (Wakai et al., 2004). A sulfur oxygenase reductase (SOR) catalyzes the disproportionation of elemental sulfur to produce sulfite, thiosulfate, and sulfide (Kletzin, 1989, 1992). A sulfur dioxygenase (SDO) can oxidize the thiol-bound sulfane sulfur atoms (R-S-SH) which is activated from S_8_ (Rohwerder and Sand, 2003, 2007). It was proposed that the disulfide reductase complex (HdrABC) could catalyze sulfane sulfate (RSSH) to produce sulfite and regenerate RSH, following donation of electrons to the quinone pool (Quatrini et al., 2009). The Rhodanese (RHD) enzyme can transfer a sulfur atom from thiosulfate to sulfur acceptors such as cyanide and thiol compounds (Schlesinger and Westley, 1974; Gardner and Rawlings, 2000). Furthermore, two thiosulfate-transferring proteins, DsrE and TusA, react with tetrathionate to yield protein Cys-S-thiosulfonates, and trigger an irreversible transfer of thiosulfate from DsrE to TusA. This indicates that both these proteins are important players in the dissimilatory sulfur and tetrathionate metabolism (Liu et al., 2014). The *tetH* cluster of *A. caldus* includes *ISac*1, *rsrR, rsrS, tetH*, and *doxD*, which encode a transposase, a RsrS-RsrR two-component system (TCS), a tetrathionate hydrolase and a thiosulfate:quinol oxidoreduetase subunit, respectively (Rzhepishevska et al., 2007). The differences in the expression of TetH with different sulfur substrates and the location of the RsrS-RsrR system upstream of the *tetH* gene imply that a regulatory mechanism exists at the transcriptional level (Bugaytsova and Lindström, 2004; Rzhepishevska et al., 2007). However, up to now nothing is known about this potential mechanism. Additionally, we also found a σ^54^-dependent two-component system (named TspS-TspR), upstream of the *sox*-I cluster of *A. caldus* (unpublished data). The discoveries of TCSs in *tetH* and *sox* clusters of *A. caldus* indicated that TCSs are potentially involved in signal transduction from substrate sensing to subsequent transcriptional regulation of the sulfur-oxidizing genes. These TCS-dependent regulatory systems possibly allow *A. caldus* to adapt to a variety of sulfur energy sources in different growth environments.

TCSs are predominant signal transduction components used by prokaryotic microorganisms to convert rapid environmental changes into specific adaptive responses (Bourret and Silversmith, 2010; Capra and Laub, 2012; Lehman et al., 2015). They typically consist of a membrane-bound sensor histidine kinase (HK), which senses a specific environmental stimulus and undergoes autophosphorylation, and a cognate response regulator (RR), which receives the phosphoryl group via various phosphotransfer pathways and modulates gene transcription by binding to *cis* regulatory elements in the promoter region (Forst et al., 1989; Huang et al., 1997; Bilwes et al., 1999; Stock et al., 2000; Bourret and Silversmith, 2010).

The most effective way to study gene function *in vivo* is mutagenesis of the gene of interest. Gene transfer methods, conjugation, and electroporation, have been developed for *A. caldus*, and mutants were constructed by a marker replacement knockout method (Liu et al., 2007; Zyl et al., 2008; Chen et al., 2010, 2012). However, previously reported gene knockout methods are extremely difficult and not reproducible. Moreover, the antibiotic marker gene introduced into the mutants makes it difficult for creating multiple mutations and may lead to polar effects on downstream genes as well as cause potential biological safety issues in industrial applications. Therefore, the development of a reliable and markerless gene knockout method is of great significance for performing molecular biology research and genetic engineering in *A. caldus*.

In order to detect the regulatory mechanism of the S_4_I pathway, we analyzed the *tetH* cluster of the sulfur oxidation system in *A. caldus*. We developed an efficient markerless gene knockout system for *A. caldus* and successfully obtained knockout mutants of *rsrR* and *rsrS*. Physiological and transcriptional analyses of the mutants were carried out to uncover the regulatory mechanism of RsrS-RsrR on the S_4_I pathway.

## Materials and methods

### Bacterial strains and growth conditions

The bacterial strains and plasmids used in this study are listed in Table 1. The strain *A. caldus* MTH-04 has been deposited in the China General Microbiological Culture Collection Center (CGMCC) with the accession number CGMCC 1.15711. Liquid Starkey-S^0^ and -K_2_S_4_O_6_ inorganic media and solid Starkey-Na_2_S_2_O_3_ plates for *A. caldus* MTH-04 culture were prepared as reported previously (Jin et al., 1992). Elemental sulfur (S^0^; boiling sterilized, 8g/L) or K_2_S_4_O_6_ (membrane filtration, 3 g/L) were added prior to inoculation. Chloromycetin, kanamycin, and streptomycin were added to a final concentration of 34, 100, and 100 μg/mL in LB media, and at 60, 100, and 100 μg/mL in liquid and solid Starkey media. The culture conditions were 37°C, 200 r/min for *Escherichia coli* (*E. coli*), and 40°C, 150 r/min for *A. caldus* MTH-04 (Chen et al., 2012).

**Table 1:** **Strains and plasmids used in the study**.

**Strains or plasmids**	**Genotype or description**	**Source or references**
**STRAINS**		
*A. caldus* MTH-04	Isolated from Tengchong area, Yunnan province, China	Our lab
*A. caldus* MTH-04 Δ*rsrR* mutant	Δ*rsrR*	This study
*A. caldus* MTH-04 Δ*rsrS* mutant	Δ*rsrS*	This study
*Escherichia coli* DH5α	${\text{F}}_{\Phi }^{-}$80d *lacZ*ΔM15Δ*(lacZYA-argF)* U169 *end A1 recA*1 *hsdR*17*(rk*^−^*,mk^+^) supE*44λ*-thi-1 gyr*96 *relA*1 *phoA*	TransGen Biotech Corp. China
*Escherichia coli* SM10	*Thr leu hsd recA* Km^r^RP4-2-Tc::Mu	Simon et al., 1983
*Escherichia coli* BL21 (DE3)	F^−^*ompT hdsSB*(Rb^−^mB^−^) *gal dgmmet*(DE3)	TransGen Biotech Corp. China
**PLASMIDS**		
pMD19-T	Ap^r^, ColE1 replicon	TaKaRa Cor.
pSDUDI	suicide plasmid; Ap^r^; Km^r^; oriTRP4; multi-cloning sites	This study
pSDUDI::rsrR(UHA + DHA)	suicide plasmid for *rsrR* deletion	This study
pSDUDI::rsrS(UHA + DHA)	suicide plasmid for *rsrS* deletion	This study
pSDU1-tac	Cm^r^; IncQ, mob^+^, tac promoter	Our lab
pSDU1-I-SceI	Cm^r^; mob^+^; Ptac; containing I*-Sce*I gene	This study
pET-22b	Amp^r^	Novagen Cor.
pJRD215	Sm^r^, Km^r^; IncQ, Mob^+^	Davison et al., 1987
pJRD-P360IRS	Sm^r^, Km^r^; IncQ, Mob^+^; PtetH(360 bp); *gusA*	This study
pJRD-P148IRS	Sm^r^, Km^r^; IncQ, Mob^+^; PtetH(148 bp); *gusA*	This study
pJRD-P90	Sm^r^, Km^r^; IncQ, Mob^+^; PtetH(90 bp); *gusA*	This study
RP4	Ap^r^, Tc^r^, Km^r^; IncP, tra^+^	Datta et al., 1971
pACBSR	Cm^r^; I-*Sce* I, λ-Red recombination system	Herring et al., 2003

The wild type and mutant strains of *A. caldus* were initially grown on a solid Starkey-Na_2_S_2_O_3_ plate. One colony from each plate was inoculated into 10 mL Starkey-S^0^ liquid medium and grown to stationary phase. The saturated 10 mL culture was then transferred to 150 mL Starkey-S^0^ liquid medium and allowed to grow to stationary phase. Finally, cells in the 150 mL culture were collected by centrifugation at 12000 × g for 5 min and diluted with Starkey liquid medium to a final concentration of OD_600_ = 1.0. In order to measure growth or extract RNA, 1 mL of this culture was inoculated into 150 mL Starkey-S^0^ or -K_2_S_4_O_6_ liquid medium. Cell growth in Starkey-S^0^ medium was measured at OD_600_ after removal of elemental sulfur in the sample by low-speed centrifugation at 400 × g for 5 min (Yu et al., 2014). Only a small amount of cells (<1.5 %) were found attached to the sulfur particles, which were neglected in the cell growth measurement. All experiments were performed in triplicate.

### Construction of plasmids pSDUDI and pSDU1-I-Sce I

To construct the basic suicide plasmid vector pSDUDI, the *ori*T region was initially amplified from the plasmid RP4 using primers oriT EcoR sen and oriT Sal ant and digested with *Eco*R I/*Sal* I. A ColE1-AmpR fragment was amplified by PCR from pMD19-T vector using primers pMD19 Sal sen and pMD19 EcoR ant and digested with *Eco*R I/*Sal* I. The two PCR products were ligated together to generate plasmid pMD-oriT. The plasmid pMD-oriT was then amplified by PCR using primers pMD19 Nde sen and oriT Apa ant to generate a linearized plasmid. This was digested with *Nde* I/*Apa* I, and ligated to *Nde* I/*Apa* I digested Km resistant gene amplified from plasmid pJRD215 using primers Km Apa sen and Km Nde ant. The resulting plasmid was the basic suicide plasmid pSDUDI. An I-*Sce* I recognition site (5′-TAGGGATAACAGGGTAAT-3′) and multiple cloning sites (MCS) were introduced into pSDUDI by PCR using primers Km Apa sen and pMD19 Nde sen/Km Nde ant, respectively. All primers used are listed in Table 2.

**Table 2:** **Primers used in construction of suicide plasmid and I-Sce I-expressing plasmid**.

**Primer name**	**Primer Sequence (5′ → 3′)**
oriT EcoR sen	ATTCCGGAATTCGCTCGTCCTGCTTCTCTTCG
oriT Sal ant	TCACGCGTCGACCGGGATTCAACCCACTCG
pMD19 Sal sen	TCACGCGTCGACGCGGTAATACGGTTATCCACAG
pMD19 EcoR ant	ATTCCGGAATTCAATGGTTTCTTAGACGTCAGGT
pMD19 Nde sen	TTATCATATGCAATTGAAGCTTGGTACCGCGGCTAGCGG
	CCGCGCGGTAATACGGTTATCCACAG
oriT Apa ant	TATAGGGCCCCGACCGGGATTCAACCCA
Km Apa sen	GTGAGGGCCCATAGGGATAACAGGGTAATAT GAATGTCA
	GCTACTGG
Km Nde ant	TTATCATATGACGCGTGGATCCGAGCTCTAGACTAG
	TCGACAATCGAAATCTCGTGATGG
pSDU1 Xba sen	CTTCATGCATTCTAGATCATGTTTGACAGCTTATC
tac Bam ant	TCGCGGATCCTCCTGCAGTGTTTCCTGTGTGAAATTG
I-Sce Bam sen	TTACGCGGATCCAGGAGGGTACCTATATGCATATGAAAAA
	CATCAAAAAAAACC
I-Sce Xba ant	TTCTTCTCTAGAACGTCGGGCCCTTATTTCAGGAAAGTTT
	CGGAGGAG

Restriction sites were indicated with underline.

The I-Sce I-expressing plasmid pSDU1-I-Sce I was constructed by generating a linearized form of the vector pSUD1-tac by PCR amplification with pSDU1 Xba sen and tac Bam ant. The I-Sce I gene was then amplified from plasmid pACBSR using primers I-Sce Bam sen and I-Sce Xba ant, digested with *Bam*H I and *Xba* I, and ligated into the *Bam*H I/*Xba* I treated linearized vector pSUD1-tac. The resulting plasmid was designated as pSDU1-I-Sce I.

### Generation of knockout mutants

To generate the suicide plasmid for *rsrR*, the upstream and downstream homologous arms (UHA and DHA) of this gene were amplified using the primer pairs R1F/R1R and R2F/R2R, respectively. The two homologous arms were then linked using fusion PCR (Yon and Fried, 1989). Finally, the fused fragments were digested with *Spe* I and *Not* I, and ligated to Spe I/Not I digested plasmid pSDUDI, thus generating the suicide plasmid pSDUDI::rsrR (UHA + DHA).

The suicide plasmid for *rsrS* was constructed in a similar manner by amplifying the two homologous arms (UHA and DHA) of *rsrS* using S1F/S1R and S2F/S2R. The UHA, DHA, and plasmid pSDUDI were then digested with *Spe* I/*Hin*d III, *Hin*d III/*Kpn* I, and *Spe* I/*Kpn* I, respectively. The three digested fragments were finally ligated to each other, and transformed into *E. coli* DH5α and screened for the suicide plasmid pSDUDI::rsrS (UHA + DHA).

The suicide plasmids for both genes were verified by restriction enzyme digestion and sequencing.

The suicide plasmids were transformed into *E. coli* SM10, and the transformed *E. coli* SM10 were conjugated with *A. caldus* as described earlier (Liu et al., 2007). After the first homologous recombination, the single crossover mutants were selected for kanamycin resistance on Starkey-Na_2_S_2_O_3_ solid plates. Single recombination events were rapidly identified by PCR using R4F/R and S4F/R primers for *rsrR* and *rsrS*, respectively. The I-*Sce* I-expressing plasmid **(**pSDU1-I-Sce I) was then transferred into the single crossover mutants to induce a second homologous recombination, thereby generating the target mutants. The Δ*rsrR* and Δ*rsrS* strains were identified by PCR based screening using R4F/R and S4F/R primers. Finally, the PCR fragments amplified from Δ*rsrR* and Δ*rsrS* were sequenced using primers R5F/R and S5F/R, respectively to confirm their identity. All primers used in this section are listed in Table S1.

Elimination of the I-*Sce* I expression plasmid was achieved by spontaneous loss. The mutant containing I-*Sce* I expression plasmid was inoculated into the non-selective liquid Starkey-S^0^ medium and grown to stationary phase. An aliquot from the culture was diluted and plated on non-selective solid Starkey-Na_2_S_2_O_3_ medium. Colony PCR was carried out to screen for loss of the plasmid using the primers RepA sen and RepC, which are also listed in Table S1. About 3–5 consecutive transfers were carried out to obtain the Δ*rsrR* and Δ*rsrS* mutants without the I-*Sce* I expression plasmid.

### Real-time quantitative PCR

For RNA isolation, the culture was filtered through filter papers with a pore size of 15–20 μm to remove the sulfur powder, after which the cells were harvested by centrifugation at 12000 × g under refrigerated conditions. RNAprotect Bacteria Reagent (Qiagen Cor.) was added to resuspend the cells and inhibit changes to RNA transcripts. The suspension was mixed immediately by vortexing for 5 s, incubated for 5 min at room temperature, and centrifuged for 10 min at 5000 × g. The supernatant was discarded and the harvested cells were stored at −70°C. The RNAs were extracted by using RNAiso Plus kit (TaKaRa Cor.) according to the manufacturer's instructions. Reverse transcription was performed using the PrimeScript^TM^ RT reagent Kit with gDNA Eraser (Perfect Real Time) (TaKaRa Cor.). One microgram of total RNA was used for every 20 μL reverse transcription reaction system to obtain cDNA under the following reaction conditions: 42°C for 2 min, 37°C for 15 min, and 85°C for 5 s. The cDNAs from various cultures were used with SYBR® *Premix Ex Taq*^TM^ (TliRNaseH Plus; TaKaRa Cor.) for real-time quantitative PCR reactions, which were performed using LightCycler® 480 (Roche). Two-hundred nanograms of cDNA was used in a 20 μL RT-qPCR reaction. The conditions for qPCR were as follows: 95°C for 30 s followed by 40 cycles at 95°C for 5 s and 60°C for 30 s, and a final cycle at 95°C for 5 s, 60°C for 1 min and 95°C with continuous mode. The data and fold change were calculated using the LightCycler® 480 software. The primers used in this assay were designed using PRIMER PREMIER 5 software (PREMIER Biosoft Int., Palo Alto, CA, USA) and are listed in Table S2. The *gapdH* gene of *A. caldus*, encoding glyceraldehyde-3-phosphate dehydrogenase, was used as the reference gene for normalization (Livak and Schmittgen, 2001). Relative expression was calculated using the comparative ΔΔC_T_ method, and the values were expressed as 2^−ΔΔCT^ (Livak and Schmittgen, 2001). Three independent replicates were performed for each experiment. The values shown in this study are the means of three independent replicates showing fold changes (FC). FC ≥ 2, *P* ≤ 0.05 and FC ≤ 0.5, *P* ≤ 0.05 were regarded as significant changes, designated as up-regulation and down-regulation, respectively. No significant change was inferred when 0.5 ≤ FC ≤ 2, *P* ≥ 0.05. The *SD*-value was calculated using Origin software with “descriptive statistics,” and the *P*-value was calculated by using GraphPad Prism software with “unpaired *t*-test.”

### EMSA assays

The expression and purification of RsrR was performed as described. The *rsrR* gene of *A. caldus* MTH-04 was amplified by PCR using primers rsrR-F and rsrR-R listed in Table S3. The PCR product was cloned into plasmid pET-22b, generating the expression plasmid pET-22b-rsrR. A positive clone was verified by sequencing, and transformed into *E. coli* BL21 (DE3). RsrR was purified using HisTrap HP column (GE Healthcare), and the concentration of the purified protein was determined using the Bradford assay.

DNA fragments were obtained by PCR amplification using different sets of primers listed in Table S3. The G360, T360, T148, and T90 fragments were obtained using primer-pairs G360-F and G360-R, T360-F and T360/148/90-R, T148-F and T360/148/90-R, and T90-F and T360/148/90-R, respectively. The G360+58IRS fragment was obtained after two rounds of PCR. The first round of PCR was performed with G360 fragment as the template using the primer-pair G360-F and G360+58IRS–R1. In the second round of PCR, the PCR product from the first round was used as the template with the primer-pair G360-F and G360+58IRS–R2, to generate the G360+58IRS fragment. The T360Δ19 fragment was obtained by fusion PCR. Two fragments were generated by PCR from the T360 fragment using the primer-pairs T360-F and T360Δ19-R, and T360Δ19-F1 and T360/148/90-R. Fusion PCR was then performed using the two generated fragments as templates with the primer pair T360-F and T360/148/90-R to obtain the T360Δ19 fragment without the 19 bp IRS. After PCR amplification, the amplified fragments were purified using QIAquick Gel Extraction Kit (Qiagen Corp.), desalted, and concentrated using ultrafiltration. Ultrafiltration was carried out using Amicon Ultra-15 mL, 3 kDa Centrifugal Filter Unit (Millipore Corp.) at 5000 × g in a refrigerated centrifuge.

EMSA assays were performed as described in Pardo and Orejas (2014). Initially, a 15 μL reaction mixture containing 1.5 μL 10 × Binding Buffer, 1 μg of salmon sperm DNA, and 1 μg RsrR was mixed and used as the reaction solution. Reaction solution containing PBS (phosphate buffer saline) instead of RsrR was used as a negative control. Both solutions were prepared and incubated at room temperature for 20 min. Two-hundred nanogram of DNA fragments were then added to both mixtures and incubated at room temperature for another 20 min. The DNA-protein complexes in both reactions were separated on 6.5% nondenaturing polyacrylamide gels in 0.25 × TBE (22.25 mM Tris-Boric acid, 0.5 mM EDTA) on ice (150 volts for 1.5 h). Visualization of the bands was done using ethidium bromide staining as described earlier (Bidart et al., 2014).

The primers used for constructing the three IRS-probe vectors and the primers that were used to verify the function of the 19bp-IRS *in vivo* are listed in Table S4.

### Gene sequences

The nucleotide sequences of *rsrR* and *rs*r*S* have been deposited with GenBank accession numbers KX161704 and KX161705, respectively. This Whole Genome Shotgun project for *A.caldus* MTH-04 has been deposited at DDBJ/ENA/GenBank under the accession LXQG00000000.

## Results

### Analysis of *tetH* clusters in *A. caldus* and other sulfur oxidizers

**Figure 1:**
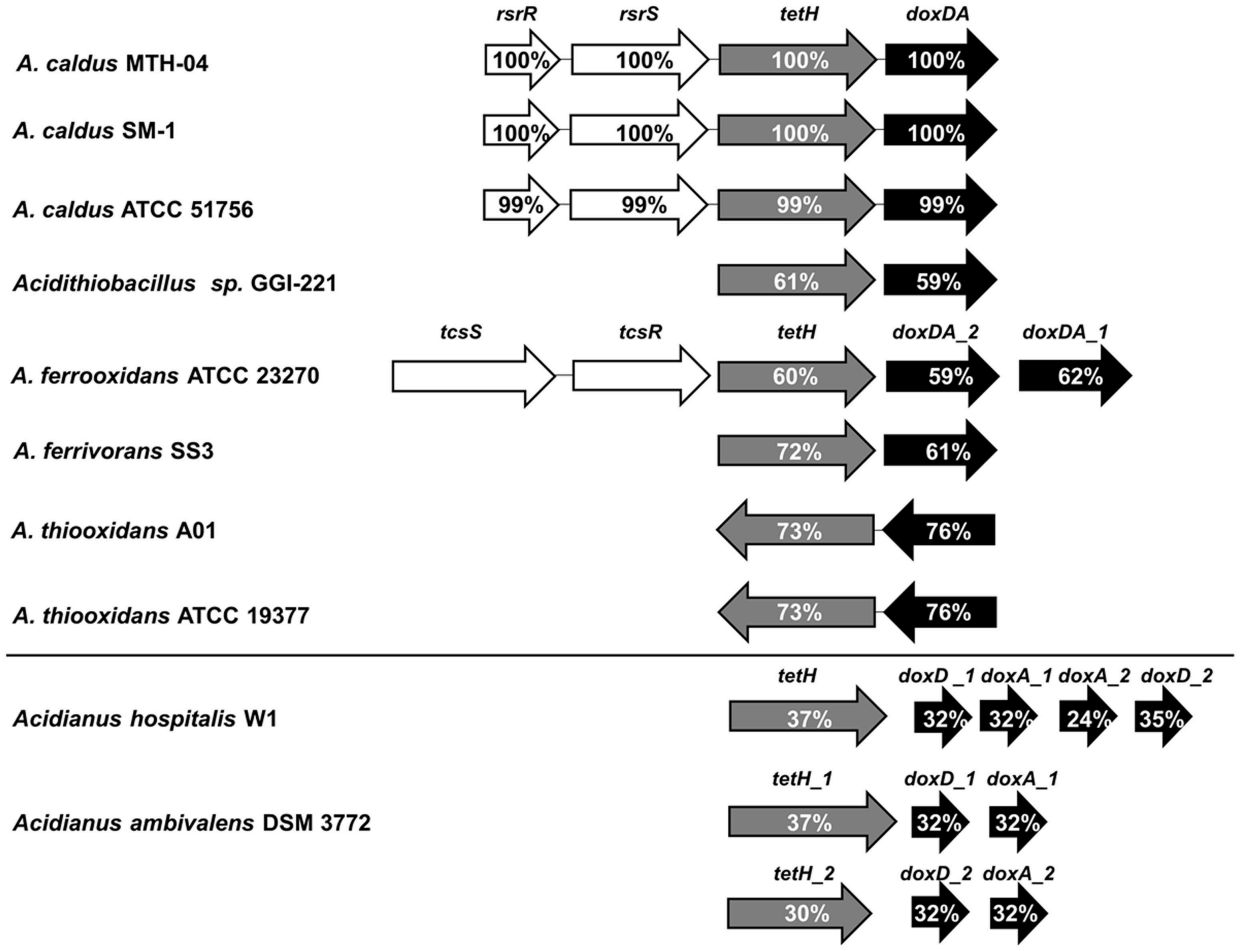
**Comparison of *tetH* clusters in various bacteria and archaea**. *rsrR* and *tcsR*, response regulators of TCSs (two-component systems); *rsrS* and *tcsS*, histidine kinases of TCSs; *tetH*, tetrathionate hydrolase; *doxDA*, thiosulfate:quinone oxidoreductase. The percentages of amino acid similarity between proteins and their homologs are indicated. The database of National Center for Biotechnology Information (NCBI), http://www.ncbi.nlm.nih.gov/ was used in this study. NCBI Taxonomy IDs for *Acidithiobacillus caldus* SM-1, *Acidithiobacillus caldus* ATCC 51756, *Acidithiobacillus* sp. GGI-221, *Acidithiobacillus ferrooxidans* ATCC 23270, *Acidithiobacillus ferrooxidans* SS3, *Acidithiobacillus thiooxidans* A01, *Acidithiobacillus thiooxidans* ATCC 19377, *Acidianus hospitalis* W1, and *Acidianus ambivalens* DSM 3772 are 990288, 637389, 872330, 243159, 743299, 1432062,637390, 933801, and 2283, respectively. Accession numbers (GenBank) for these proteins are: *A. caldus* MTH-04, RsrR (ANJ65973.1), RsrS (ANJ65974.1), TetH (OAN03451.1), DoxDA (OAN03452.1); *A. caldus* SM-1, RsrR (AEK59530.1), RsrS (AEK58242.1), TetH (AEK58243.1), DoxDA (AEK58244.1); *A. caldus* ATCC 51756, RsrR (ABP38227.1), RsrS (ABP38226.1), TetH (ABP38225.1), DoxDA (ABP38224.1); *Acidithiobacillus sp.* GGI-221, TetH (EGQ60847.1), DoxDA (EGQ62792.1); *A. ferrooxidans* ATCC 23270, TcsS (WP_041646693.1), TcsR (WP_012535753.1), TetH (ACK80599.1), DoxDA_2 (ACK79881.1), DoxDA_1 (ACK78481.1); *A. ferrooxidans* SS3, TetH (AEM46280.1), DoxDA (AEM47534.1); *A. thiooxidans* A01, TetH (WP_024894935.1), DoxDA (WP_024894934.1); *A. thiooxidans* ATCC 19377, TetH (WP_029316048.1), DoxDA (WP_010638552.1); *Acidianus hospitalis* W1, TetH (AEE94548.1), DoxD_1 (AEE93006.1), DoxA_1 (AEE93005.1), DoxA_2 (AEE93131.1), DoxD_2 (AEE93130.1); *Acidianus ambivalens* DSM 3772, TetH_1 (CBY66038.1), DoxD_1 (CAA69986.1), DoxA_1 (CAA69987.1), TetH_2 (CBY66040.1), DoxD_2 (CAA70827.1), DoxA_2 (CAA70828.1).

The *tet*H clusters in various bacteria and archaea were compared for analysis. As shown in Figure 1, the two functional genes (*tetH* and *doxDA*) of the S_4_I pathway were distributed in chemoautotrophic sulfur oxidizers including bacteria and archaea of the genera *Acidithiobacillus* and *Acidianus*, respectively. However, only *A. caldus* and *Acidithiobacillus ferrooxidans* have a specific two-component system (TCS) upstream the *tetH* gene. The cluster of *A. caldus* MTH-04 was identical to that of *A. caldus* SM-1 and shared 99% identity with that of *A. caldus* ATCC 51756. Furthermore, the *tetH* cluster in *Acidithiobacillus* sp. GGI-221 has certain components including *tetH* and *doxDA* with 61 and 59% identity, respectively. The 16S rRNA gene sequence of *Acidithiobacillus* sp. GGI-221 shows 99.7% identity to *Acidithiobacillus ferrooxidans* indicating that this is a strain of *Acidithiobacillus ferrooxidans* (Williams and Kelly, 2013). However, the TCS of *A. ferrooxidans* is σ^54^-dependent and has a different order from *tcsS* to *tcsR* encoding the sensor histidine kinase and the cognate response regulator when compared to *A. caldus.* The *tetH* and *doxDA* genes are arranged in a cluster in *A. caldus* and *Acidithiobacillus thiooxidans*, while they were separately distributed in the genomes of *Acidianus* and other *Acidithiobacillus* spp. Two copies of *doxDA* genes are located separately in the genomes of *A. ferrooxidans* and *Acidianus hospitalis* W1, while in *Acidianus ambivalens* DSM 3772 there are two copies of *tetH*. Moreover, *doxDA* in *Acidithiobacillus* spp. encodes a protein with two domains DoxD and DoxA, but DoxD and DoxA in *Acidianus hospitalis* W1 and *Acidianus ambivalens* DSM 3772 are two individual subunits encoded by two separate genes, *doxD* and *doxA* (Müller et al., 2004; Valenzuela et al., 2008; Valdés et al., 2009; Mangold et al., 2011). BLAST and multiple alignment (Figure S1) results demonstrated that the two subunits DoxD and DoxA are fused as one protein in the three *A. caldus* strains, MTH-04, SM-1, and ATCC 51756, indicating that the *doxD* gene in this *tetH* cluster encodes a protein that has two domains corresponding to DoxD and DoxA (Müller et al., 2004). Thus, we renamed the *doxD* gene in these strains as *doxDA* or *tqo*.

### Development of a markerless gene knockout system to construct Δ*RsrS* and Δ*RsrR*

**Figure 2:**
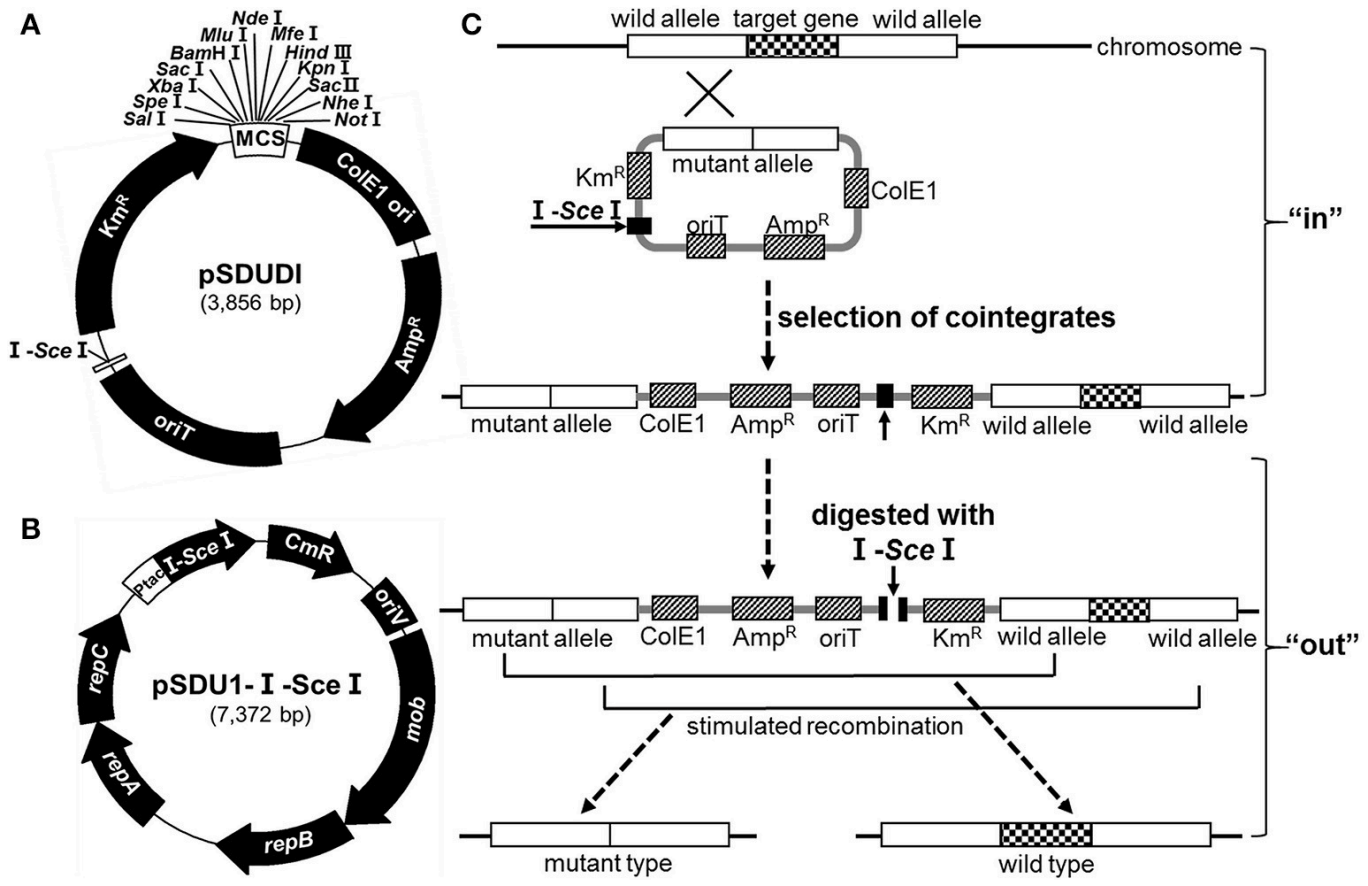
**The markerless gene knockout strategy in *A. caldus*. (A)** Map of the suicide plasmid pSDUDI. ColE1, the replication origin; *ori*T, the origin of transfer; I-SceI, endonuclease recognition site. **(B)** Plasmid pSDU1-I-Sce I. I-Sce I endonuclease gene was under the control of Ptac promoter. **(C)** General scheme of the markerless gene knockout method. At the “in” step, the suicide plasmid carrying homologous sequences specific to the targeted gene was conjugated into *A. caldus*, and integrated into the chromosome with the help of Rec homologous recombination system in *A. caldus*. At the “out” step, the I-Sce I endonuclease encoded by plasmid pSDU1-I-Sce I created the double-stranded breaks (DSBs) on the chromosome, which acted as the signal to stimulate a second recombination event, generating either a wild type or a mutant type.

A mobile suicide vector (pSDUDI, Figure 2A) was employed to introduce homologous sequences of the target genes into *A. caldus* cells, and to integrate these homologous sequences into the genome by homologous recombination, thus generating cointegrates (Figure 2C). The backbone of the suicide plasmid was derived from pUC19 and therefore cannot replicate in *A. caldus*. Second, the origin of transfer (*ori*T) of plasmid RP4 was cloned into this plasmid, which allows it to be mobilized into *A. caldus* with high efficiency. Third, an 18 bp I-*Sce* I endonuclease recognition site (5′-TAGGGATAACAGGGTAAT-3′) was also introduced into this plasmid to facilitate cleavage of the cointegrate by I-Sce I endonuclease.

The I-*Sce* I-expressing plasmid **(**pSDU1-I-Sce I) shown in Figure 2B was derived from pJRD215, which can replicate and remain stable in *A. caldus*. The Ptac promoter was introduced into this plasmid to express I-*Sce* I in *A. caldus*. Mobilization of pSDU1-I-Sce I into the cointegrate of *A. caldus* would lead to the generation of double-stranded breaks (DSBs) at the I-*Sce* I site. The subsequent second homologous recombination event, would ultimately lead to generation of the mutant or wild type strains (Figure 2C).

**Figure 3:**
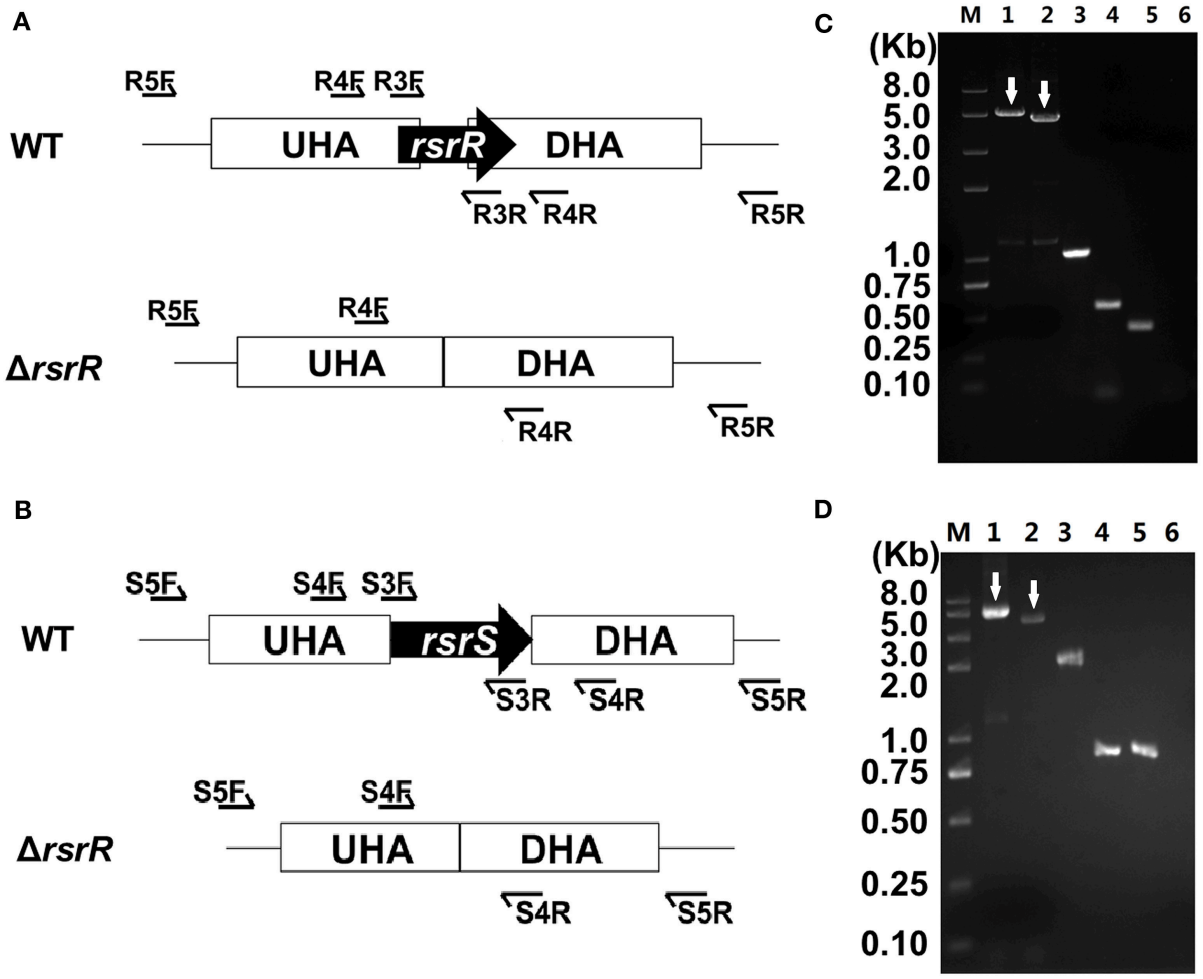
**Confirmation of *rsrR* and *rsrS* mutants by PCR analyses. (A,B)** Diagram of three sets of primers specific to target genes (*rsrR* and *rsrS*), the upstream and downstream homologous arms (UHA and DHA) and the sequences outside of homologous arms, respectively. **(C,D)** PCR analyses of the chromosomes of Δ*rsrR* and Δ*rsrS*. Lane 1 and 2, PCR amplifications from wild type and mutants using primers R5F/R or S5F/R, respectively; lane 3 and 4, PCR amplifications from wild type and mutants using primers R4F/R or S4F/R, respectively; lane 5 and 6, PCR amplifications from wild type and mutants using primers R3F/R or S3F/R, respectively. In **(C,D)**, the arrows are added to indicate the target bands.

Verification of the Δ*rsrR* and Δ*rsrS* strains is shown in Figure 3. Smaller fragments were amplified from Δ*rsrR* and Δ*rsrS* strains compared to the wild type using primers R4F/R and S4F/R (lane 1, 5.1 kb; lane 2, 4.6 kb; lane 3, 1.0 kb, and lane 4, 551 bp), and R5F/R and S5F/R (lane 1, 5.0 kb; lane 2, 3.9 kb; lane 3, 2.1 kb, and lane 4, 910 bp; Figures 3C,D lanes 1–4). No band could be amplified from *rsrR* and *rsrS* knockout strains using primers R3F/R and S3F/R (Figures 3C,D lanes 6), while there were 413 and 850 bp bands for *rsrR* and *rsrS* genes, respectively in the wild type as shown in Figures 3C,D lanes 5. The sizes of the observed PCR bands were as expected in different strains. The precise sequences of the two mutants were confirmed by sequencing the PCR fragments (Figures 3C,D lane 2) derived from Δ*rsrR* and Δ*rsrS*.

### Transcriptional changes of the *tetH* cluster

**Figure 4:**
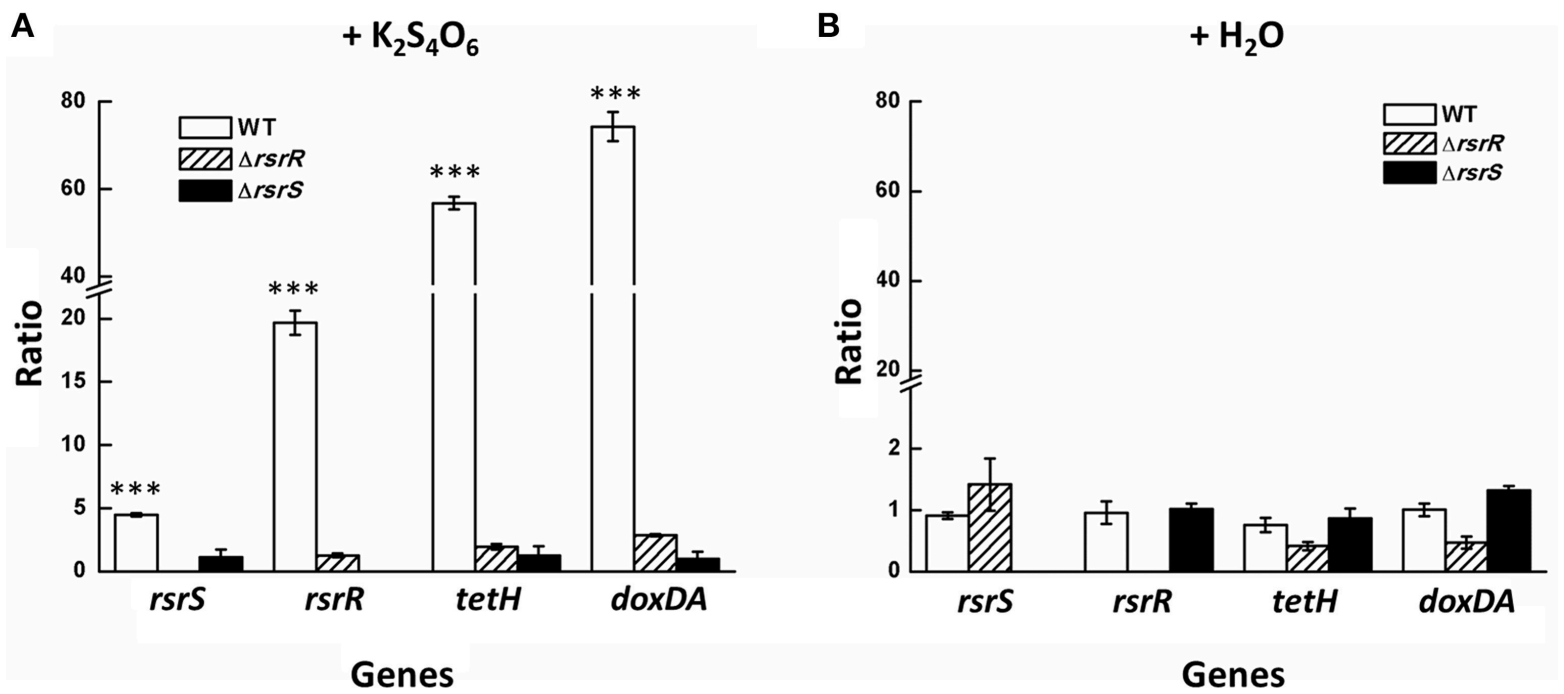
**Relative RNA transcripts level changes of *tetH* cluster in Δ*rsrR*, Δ*rsrS*, and the wild type in S^0^-medium with the addition of K_2_S_4_O_6_ (A) and H_2_O (B)**. The addition of the same volume H_2_O was set as the control group. Asterisks denote statistically significant changes (^***^P ≤ 0.001).

To verify the influence of the lack of RsrR or RsrS on the S_4_I pathway, relative RNA transcript levels of genes in the *tetH* cluster of Δ*rsrR*, Δ*rsrS*, and the wild type strains were tested by RT-qPCR. Strains were grown to mid-log phase in S^0^-medium (at the 4th day), followed by addition of equal volumes of either K_2_S_4_O_6_ or H_2_O to the stimulation and control groups, respectively. Cells at the 4th and 4.5th days were collected to purify total RNA for transcriptional analysis. The ratio of relative RNA transcripts levels of the genes at the two time points were calculated and are shown in Figure 4. After stimulation with exogenous K_2_S_4_O_6_ at a final concentration of 3 g/L, the relative RNA transcript levels of *rsrS, rsrR, tetH*, and *doxDA* in the wild type increased by about 5, 20, 60, and 80 fold, respectively (Figure 4A). However, the relative transcript levels of these genes in the two mutants did not show any obvious increase (0.5 ≤ ratio ≤ 2.0; Figure 4A). In the control group, addition of water did not result in a significant change in expression of any of the four genes in the three strains (0.5 ≤ ratio ≤ 2.0; Figure 4B). This result supported the notion that there is a K_2_S_4_O_6_-dependent positive regulation of RsrS-RsrR on *tetH*-*doxDA*.

### Determination of the *Cis*-regulatory element of RsrR

**Figure 5:**
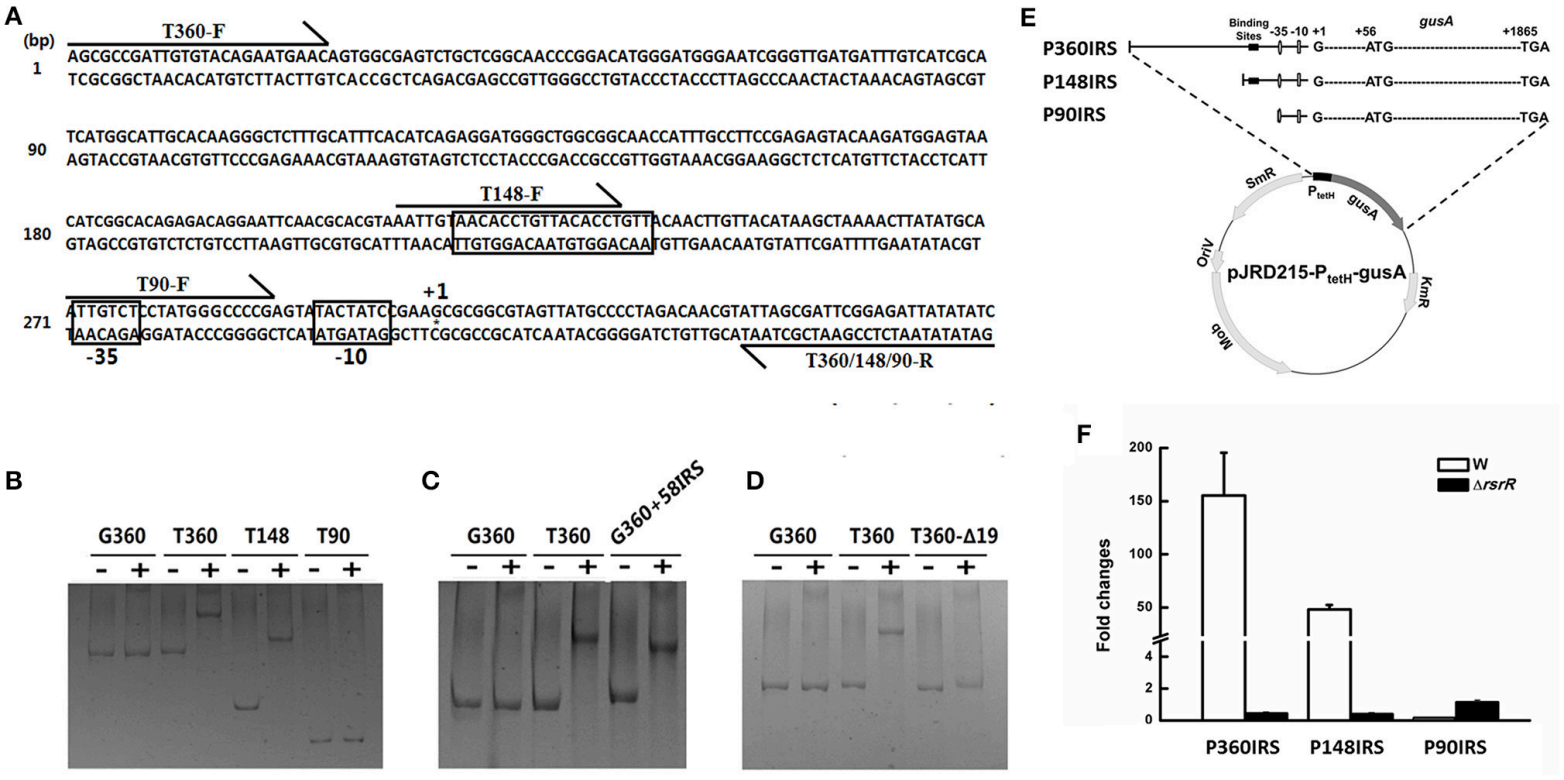
**The functional identification of a 19-bp inverted repeat sequence (IRS) upstream the *tetH* promoter. (A)** The loci of IRS, −10/−35 regions, the transcriptional start site and primers on the sequence upstream *tetH* gene. **(B–D)** The results of EMSAs to determine the binding ability of RsrR to the regulatory sequence. −, the group containing the nucleotide fragments without RsrR; +, the group containing both the different nucleotide fragments and RsrR. G360, a 360 bp-fragment from the *gapdH* gene used as a control; T360, T148 and T90, a 360 bp-, 148 bp-, or 90 bp-fragment amplified from upstream region of *tetH*; G360+58IRS, a 58 bp sequence containing the IRS fused with the 360 bp-fragment from *gapdH*; T360Δ19: the T360 fragment with IRS removed. **(E)** Diagram of series of IRS-probe vectors containing various versions of the upstream region of *tetH*. **(F)** Transcriptional analysis of *gusA* on the IRS-probe vectors in Δ*rsrR* and the wild type.

Positive control of the cotranscription of *tetH* and *doxDA* (Rzhepishevska et al., 2007) is likely achieved by binding of RsrR to a *cis*-regulatory element at the promoter region of *tetH*. To confirm the above hypothesis, three fragments about 360, 148, and 90 bp upstream of the “ATG” of *tetH* were amplified to test for their interaction with RsrR by electrophoretic mobility shift assays (EMSAs) (Figure 5A). A 360 bp fragment amplified from the *gapdH* gene of *A. caldus* was used as a negative control. The results showed that the binding region is located in a 58 bp region between 90 and 148 bp upstream of “ATG” (Figure 5B). When the 58 bp region was fused with the 360 bp *gapdH* fragment, the fusion could bind to RsrR (Figure 5C), indicating that this *cis*-regulatory element was located in the 58 bp region. To further narrow down the binding region of RsrR, the software package Repeat Around-2.1 was used to analyze this region and a 19 bp (AACACCTGTTACACCTGTT) inverted repeat sequence (IRS) within the 58 bp region was predicted to be the binding sequence (Figure 5A). Upon removal of the 19 bp-IRS from the 360 bp-fragment upstream of *tetH*, binding of RsrR could not be observed (Figure 5D), which suggested that the 19 bp-IRS was the key binding site of RsrR.

To verify the function of the 19 bp-IRS on the transcription of *tetH* and *doxDA in vivo*, the 360, 148, and 90 bp fragments upstream of the “ATG” of *tetH* were fused to the reporter gene *gusA* to generate three IRS-probe vectors, as shown in Figure 5E. The resulting plasmids were designated as pJRD-P360IRS, pJRD-P148IRS, and pJRD-P90, respectively and were mobilized into wild type and Δ*rsrR* strains. All strains were grown in S^0^-medium, and the relative RNA transcript levels of *gusA* in each strain were measured after stimulation with K_2_S_4_O_6_. As shown in Figure 5F, a significantly low level of *gusA* transcript was observed in the wild type strain harvesting plasmid pJRD-P90 when compared with that with pJRD-P360IRS and pJRD-P148IRS, indicating that the 19 bp-IRS had a positive effect on the transcription of *tetH* promoter. The relative *gusA* RNA transcript levels from plasmids pJRD-P360IRS and pJRD-P148IRS in Δ*rsrR* were much lower than those in the wild type strain, indicating that IRS is needed for the positive effect of RsrR.

Structural simulation and protein sequence alignment between RsrS-RsrR and EnvZ-OmpR were also carried out in order to help understand the mechanism of regulation of the *tetH* cluster by RsrS-RsrR, which showed that the two TCSs are highly identical at the protein level (Figure S2).

### Growth analyses of the mutants in different sulfur-substrate media

**Figure 6:**
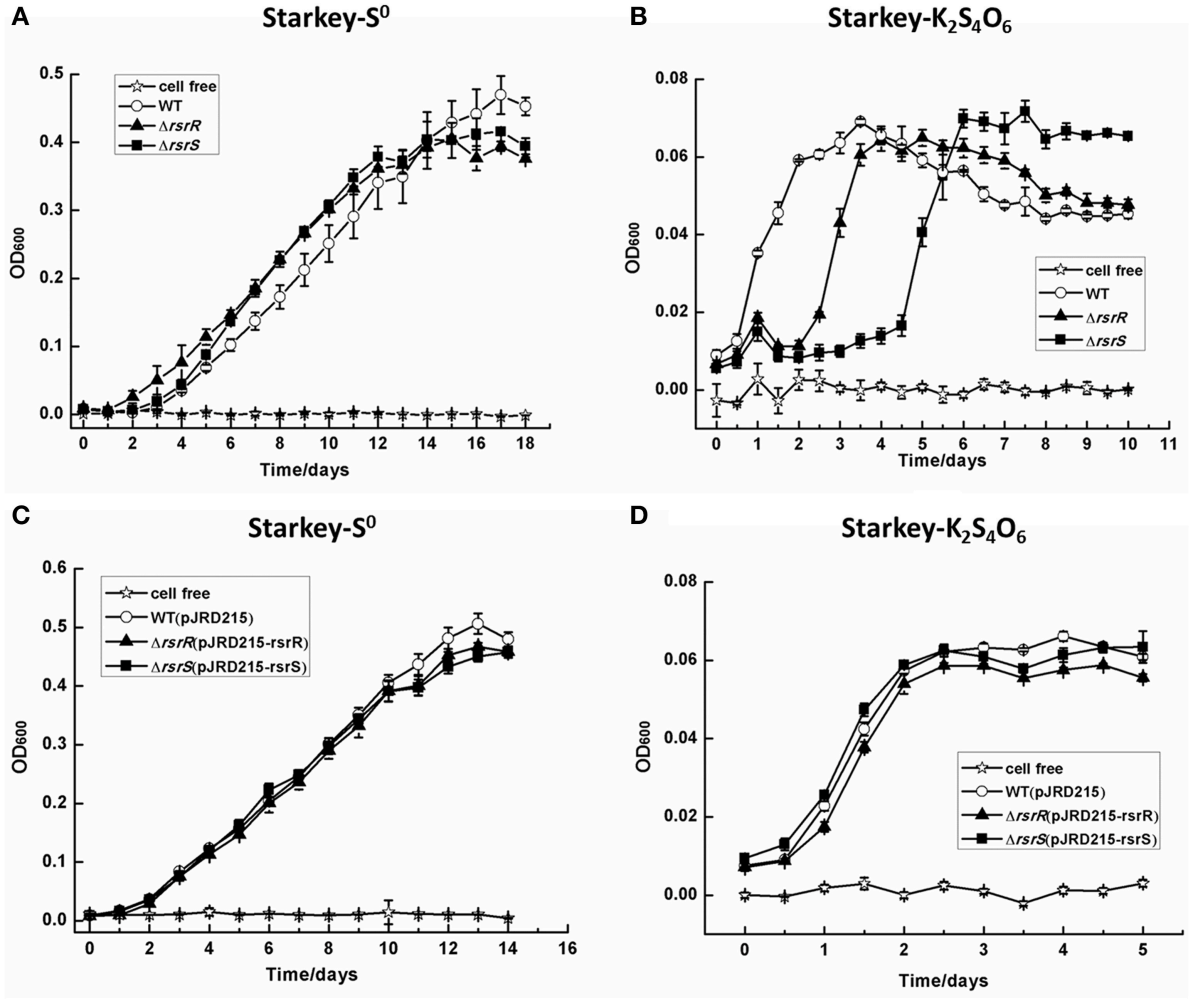
**The growth curves of the wild type, Δ*rsrR*, and Δ*rsrS* mutants, and complemented strains of *A. caldus* MTH-04 grown in Starkey medium to which were added different energy sources**. All the measurements were performed in triplicate. The values for OD600 are the mean of the three independent replicates. The *SD*-values are shown in the figure with short bars on the top of the columns, and they were calculated by using the Origin software with “descriptive statistics.” **(A,C)** Growth in Starkey-S^0^ medium; **(B,D)** Growth in Starkey-K_2_S_4_O_6_ medium. WT, Δ*rsrR*, and Δ*rsrS*, WT(pJRD215), Δ*rsrR*(pJRD215-rsrR), and Δ*rsrS*(pJRD215-rsrS) represent the wild type, mutants, wild type carrying plasmid pJRD215, and complemented strains of *A. caldus* MTH-04, respectively.

As shown in Figure 6, the growth rates of the *rsrR* or *rsrS* knockout mutants were not similar to the wild type. When grown in S^0^-medium, both Δ*rsrR* and Δ*rsrS* had a slight growth advantage over the wild type strain from the 5th to the 11th day, which corresponds to the logarithmic growth phase prior to entering into the stationary phase. In contrast, the wild type strain grew slightly better than the mutants after the 15th day (Figure 6A). When K_2_S_4_O_6_ was used as the sole sulfur substrate, the three strains showed very slow overall growth (up to 0.06) compared to S^0^-medium due to differences in RISCs metabolism for different substrates (Zyl et al., 2008; Chen et al., 2012; Zhang et al., 2014). In K_2_S_4_O_6_-medium, Δ*rsrR* and Δ*rsrS* had a 2 and 4 day growth delay in lag phase, respectively when compared to the wild type. However, the three tested strains reached approximately the same growth when they reached stationary phase (Figure 6B). In addition, complementation of the mutations with wild type alleles resulted in a growth pattern similar to the wild type in both media (Figures 6C,D). Therefore, the recovery of growth of the mutants in K_2_S_4_O_6_-medium after a delay of several days suggested that RsrS-RsrR might be the primary, but not the sole signal transduction pathway that regulates the metabolism of tetrathionate.

### Influence of *RsrR* and *RsrS* on sulfur metabolism and signaling systems

**Figure 7:**
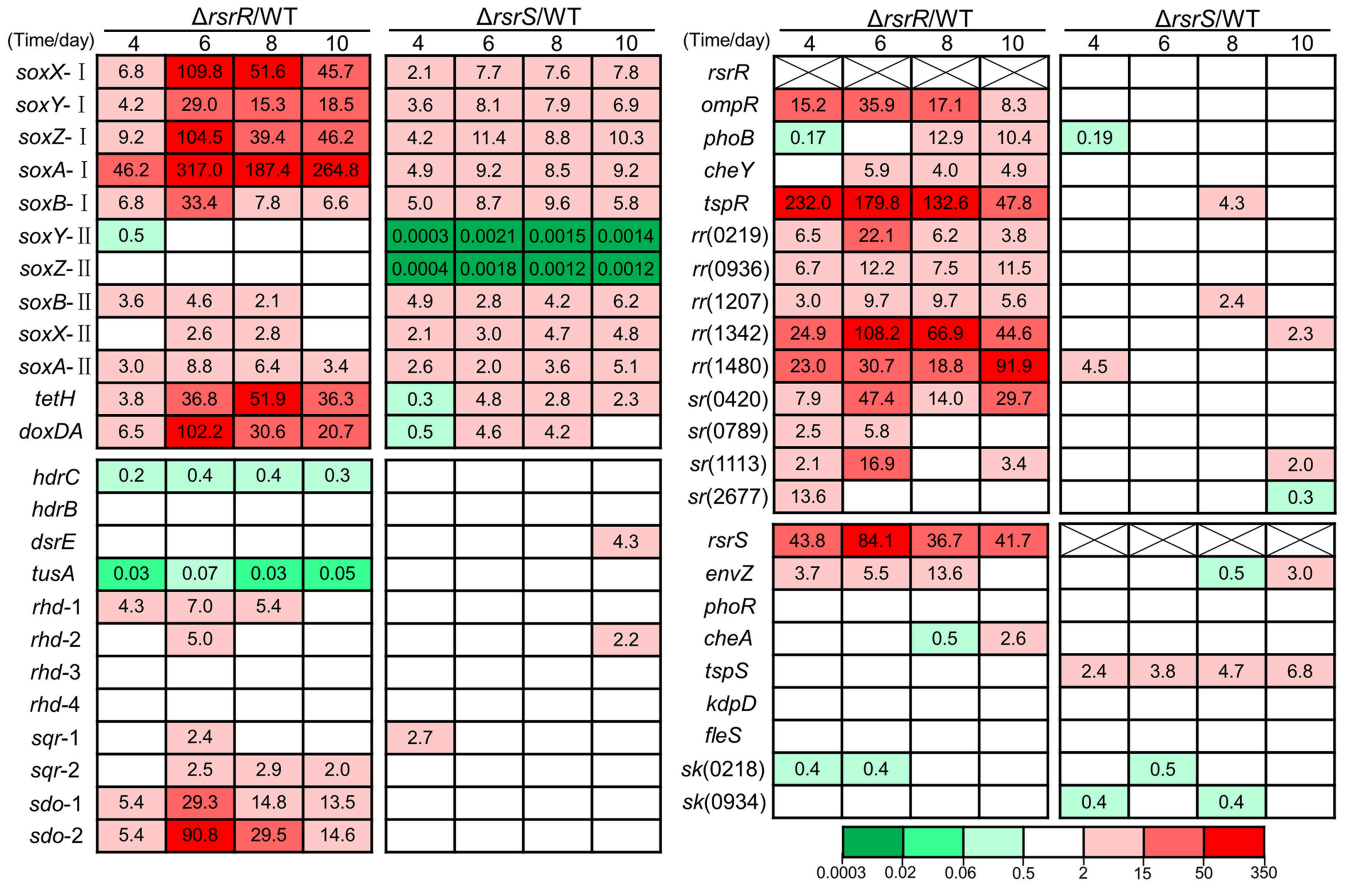
**The relative transcription levels of genes involved in sulfur metabolism and signaling systems during the S^0^-cultivating process**. This is the valid mean value of three independent replicates showing fold changes (FC) determined by RT-qPCR analyses of the mutant against the wild-type. FC ≥ 2, *P* ≤ 0.05 and FC ≤ 0.5, *P* ≤ 0.05 were regarded as the significant changes. FC ≥ 2, *P* ≤ 0.05, up-regulation; FC ≤ 0.5, *P* ≤ 0.05, down-regulation; 0.5 ≤ FC ≤ 2, *P* ≥ 0.05, no change (data are not shown in the figure). FC-values are represented with different colors as indicated in the color bar. The data of standard deviation (*SD*) and *P*-value were shown in Supplementary Text S1. The *SD*-value was calculated by using the Origin software with “descriptive statistics.” The *P*-value was calculated by using the GraphPad Prism software with “unpaired *t*-test.” The original data with the values for standard deviation and *P*-value are listed in Supplementary Text S1. The putative function of proteins encoded by these genes: *soxX*-I (A5904_2486), *soxX*-II (A5904_2525), cytochrome c class I; *soxY*-I (A5904_2487), *soxY*-II (A5904_2520), sulfur covalently binding protein; *soxZ*-I (A5904_2488), *soxZ*-II (A5904_2521), sulfur compound chelating protein; *soxA*-I (A5904_2489), *soxA*-II (A5904_2526), cytochrome c (diheme); *soxB*-I (A5904_2491), *soxB*-II (A5904_2522), sulfate thiol esterase; *hdrC* (A5904_1042), *hdrB* (A5904_1043), heterodisulfide reductase subunit C and B; *dsrE* (A5904_2473), *tusA* (A5904_2474), sulfur transferase; *rhd-1* (A5904_0894), *rhd-2* (A5904_1407), *rhd-3* (A5904_2860), *rhd-4* (A5904_2475), rhodanese (sulfur transferase); *sqr-1* (A5904_1436), *sqr-2* (A5904_2678), sulfide quinone reductase; *sdo-1* (A5904_0421), *sdo-2* (A5904_0790), sulfur dioxygenase; *envZ* (A5904_2589), *ompR* (A5904_2590), osmolarity regulation; *phoB* (A5904_0374), *phoR* (A5904_0373), phosphate regulon; *cheY* (A5904_1450), *cheA* (A5904_1448), chemotaxis; *tspR* (A5904_2485), *tspS* (A5904_2484), regulation for Sox pathway; *kdpD* (A5904_1340), unknown; *fleS* (A5904_1479), flagellum associated; *rr* (A5904_0219, A5904_0936, A5904_1207, A5904_1342, A5904_1480), putative response regulators in TCSs; *hk* (A5904_0218, A5904_0934), putative sensor histidine kinases in TCSs; *sr* (A5904_0420, A5904_0789, A5904_1113, A5904_2677), putative regulators in SCSs.

In *A. caldus*, the sulfur-oxidizing mechanisms include the periplasmic Sox system, the S_4_I pathway, and the RISCs oxidation enzymes such as SOR, SDO, SQR, HDR, RHD, DsrE, and TusA (mentioned in the Introduction Section). To investigate the effect of the absence of RsrS and RsrR on the S_4_I pathway, we analyzed the relative RNA transcript levels of genes attributed to play a role in the *A. caldus* RISCs metabolism. Cross-talk often occurs between a sensor kinase and a non-cognate response regulator in the TCSs (Procaccini et al., 2011), so genes encoding single- and two-component systems (SCSs and TCSs) were also analyzed for their relative RNA transcript levels. The transcriptional analysis of these sulfur-oxidizing and regulatory genes during cultivation on S^0^ was carried out by RT-qPCR. All data were calculated and the statistically valid data were showed as the mean value of three independent replicates in Figure 7 (original data with values for standard deviation and *P*-value are listed in Supplementary Text S1). As shown, several genes in the mutants had significant changes in expression (FC ≥ 2, *P* ≤ 0.05, up-regulation; FC ≤ 0.5, *P* ≤ 0.05, down-regulation) when compared with the wild type. The deletion of *rsrR* resulted in a sharp up-regulation (FC ≥ 6, *P* ≤ 0.05) of *tetH*-*dox*DA, *sox* operon I, *sdo*-1, and *sdo*-2, clear down-regulation (FC ≤ 0.01, *P* ≤ 0.05) of *tusA*, and weak transcriptional changes (2 ≤ FC ≤ 6, *P* ≤ 0.05) in *sox* operon II, *sqr*, and *rhd*. The absence of *rsrR* also resulted in significant up-regulation (FC ≥ 2, *P* ≤ 0.05) of a majority of regulator genes in TCSs including *ompR* (A5904_2590), *phoB* (A5904_0374), *cheY* (A5904_1450), *tspR* (A5904_2485), A5904_0219, A5904_0936, A5904_1207, A5904_1342 and A5904_1480, and in SCSs including A5904_0420, A5904_0789 and A5904_1113. The sensor histidine kinase (HK) genes *rsrS* and *envZ* in TCSs are up-regulated significantly in the *rsrR* knockout strain. However, the HK genes *phoR* (A5904_0373), *cheA* (A5904_1448), *tspS* (A5904_2484), *kdpD* (A5904_1340), *fleS* (A5904_1479), A5904_0218 and A5904_0934 had no obvious transcriptional changes in this mutant. In the Δ*rsrS* strain, the genes of the Sox operon (except *soxYZ* in operon II), *tetH*, and *doxDA* showed significant changes in expression when compared to the wild type. The expression of *soxYZ* in operon II was reduced to almost undetectable levels. Almost all the other genes involved in the sulfur oxidation system, TCSs, and SCSs did not show significant changes. However, *tspS* showed a significant change (2 ≤ FC ≤ 7, *P* ≤ 0.05) in the Δ*rsrS* strain.

## Discussion

The lack of a reliable gene knockout method for *A. caldus* has been a significant obstacle in the progress of uncovering the sulfur oxidation mechanism and other important physiological functions in this organism (Valdés et al., 2009; You et al., 2011; Chen et al., 2012). In this study, we developed a markerless gene knockout technique using a suicide plasmid and an I-*Sce* I-expressing plasmid. There are two advantages of our markerless gene knockout technique. The first is that we used an endogenous Rec (RecA and RecBCD) system of *A. caldus*, rather than an exogenous λ-Red or RecET system, for homologous recombination to avoid the incompatibility of an exogenous recombination system. The second advantage is that we used conjugation as our gene transfer method, which is the optimal way to incorporate plasmids into cells because conjugation facilitates homologous recombination between the suicide plasmid and the host chromosome (Dillingham and Kowalczykowski, 2008). Furthermore, this knockout technique can be further optimized to integrate genes or other sequences into *A. caldus*.

*A. caldus* has a complex sulfur oxidation system that includes periplasmic sulfur-oxidizing pathways (Sox and S_4_I) and cytoplasmic sulfur-oxidizing enzymes (SDO, SOR, HDR etc.) (Chen et al., 2012). The *sor* gene in the wild type and both mutants of *A. caldus* MTH-04 was lost during the long period of subcultivation in S^0^-medium under the laboratory conditions (as confirmed by sequence analysis of PCR products). The loss of *sor* is probably caused by transposition of the transposon as has been reported for the strain *A. caldus* SM-1 (You et al., 2011). The sulfur oxidation pathways in the two cellular compartments are probably connected by tetrathionate, which can enter the cytoplasm and react with DsrE or TusA to generate protein Cys-S-thiosulfonates, thus initiating sulfur metabolism in the cytoplasm (Liu et al., 2014). Comparative analysis of the S_4_I pathway genes *tetH* and *doxDA* in *Acidithiobacillus* spp. and the archaea *Acidianus hospitalis* and *Acidianus ambivalens*, indicated that only *A. caldus* evolved an *rsrRS*-*tetH*-*doxDA*-like cluster (Figure 1). The combination of two functional genes (*tetH* and *doxDA*) and the TCS regulatory genes (*rsrR* and *rsrS*) in this cluster potentially allows *A. caldus* to regulate its S_4_I pathway via the RsrS-RsrR system. Thus, *A. caldus* can efficiently maintain the balance between thiosulfate and tetrathionate in the periplasm and modulate the periplasmic and cytoplasmic sulfur-oxidizing pathways.

The positive regulatory role of RsrS-RsrR on the S_4_I pathway was inferred from the transcriptional analysis of the *tetH* cluster in the *rsrR* and *rsrS* knockout mutants and the wild type upon stimulation with K_2_S_4_O_6_. The relative transcriptional levels of genes in the *tetH* cluster in the *rsrR* or *rsrS* knockouts were much lower as compared to that in the wild type when stimulated with K_2_S_4_O_6_ (Figure 4A), indicating a positive effect of RsrS-RsrR on the transcriptional regulation of *tetH* and *doxDA*. Thus, RsrS-RsrR linked the signal from tetrathionate to the transcription of *tetH* and *doxDA*, which allowed adjustment of the S_4_I pathway in *A. caldus* to utilize tetrathionate in the growth environment.

**Figure 8:**
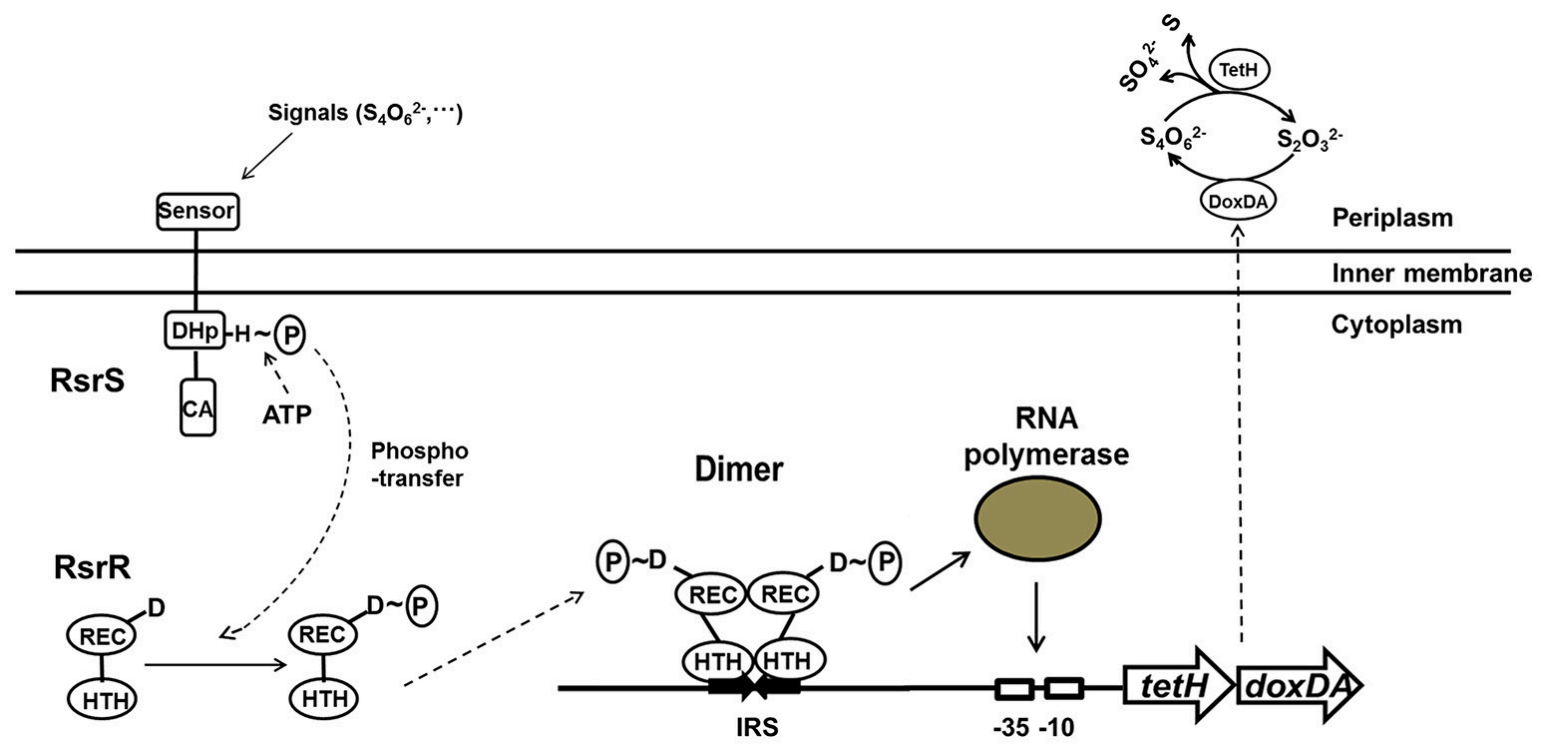
**The signal transduction and transcriptional regulation model of RsrS-RsrR on the S_4_I pathway in *A. caldus***.

The determination of the positive regulation of RsrS-RsrR for S_4_I pathway, combined with simultaneous transcription of *tetH*-*doxDA* and the P1 promoter upstream of *tetH* (Rzhepishevska et al., 2007), indicated the existence of a *cis*-regulatory element in *A. caldus*. The data from the EMSA assays *in vitro* and the promoter-probe vector analysis *in vivo* revealed direct interaction between RsrR and the IRS upstream of the *tetH* promoter, along with the effects of the IRS on the transcriptional activity of the promoter. The 19 bp-IRS (AACACCTGTTACACCTGTT) is composed of two 9 bp complementary inverted half-sites (AACACCTGT and ACACCTGTT) with a 1-bp interval (T). The RsrS-RsrR in *A. caldus* is an EnvZ-OmpR like TCS (Rzhepishevska et al., 2007), in which the response regulators (RsrR and OmpR) share 42% identity and the sensor histidine kinases (RsrS and EnvZ) share 30% identity at the amino acid level. The high level of identity in the structures and protein sequences between RsrS-RsrR and EnvZ-OmpR indicate that RsrR is a typical regulator with a winged helix-turn-helix (HTH) DNA-binding domain, allowing the RsrR dimer to interact with the 19 bp IRS through the binding of two HTH domains to the two 9 bp half-sites of the IRS. RsrS has a predicted unique sensor domain, suggesting that it has the ability to detect the signal from tetrathionate. These results are consistent with the previously reported mechanism of TCS in translating environmental stimuli to specific adaptive responses (Martínez-Hackert and Stock, 1997; Mattison and Kenney, 2002; Wang, 2012). Therefore, we propose a tetrathionate-dependent transcriptional regulation model of the S_4_I pathway by RsrS-RsrR in *A. caldus*. As shown in Figure 8, the membrane-bound sensor of histidine kinase RsrS might sense the signal from tetrathionate in the periplasm, which may then autophosphorylate the conserved His site, and transfer the phosphoryl group to the conserved Asp site of RsrR, thus generating an active dimer. The RsrR dimer might recognize and bind to the 19 bp IRS with its HTH domains to promote the transcriptional activity of the *tetH* promoter by assisting the recruitment of the RNA polymerase or by strengthening the binding between the RNA polymerase and the DNA sequence (Hochschild and Dove, 1998; Kenney, 2002).

Blocking of the signaling pathway from tetrathionate to S_4_I pathway caused changes in growth and transcription patterns. The absence of RsrS or RsrR led to several days delay of growth in K_2_S_4_O_6_-medium. The survival of mutants in K_2_S_4_O_6_-medium indicated that the RsrS-RsrR mediated signal pathway is redundant with other pathways in promoting the transcription of *tetH*-*doxDA* and decomposition of tetrathionate. Transcriptional analysis revealed that the knockout of *rsrR* had a much stronger impact on the transcription of these genes than that of *rsrS*, both in terms of the number of genes being affected and in the magnitude of changes in transcription levels. The relative change in the RNA transcript levels in the mutants during growth in S^0^-medium revealed that the knockout of either *rsrS* or *rsrR* not only caused significant up- or down-regulation of the majority of sulfur-oxidizing genes, but also resulted in significant changes in transcription of most regulatory genes. The RsrS-RsrR and EnvZ-OmpR like two-component systems share significant homology owing to their evolutionary relationship (Rzhepishevska et al., 2007). The high level of sequence similarity and close homologous relation between some TCSs raises the possibility of undesired cross-talk between a sensor kinase and a non-cognate response regulator (Howell et al., 2003; Siryaporn and Goulian, 2008; Procaccini et al., 2011; Guckes et al., 2013; Bielecki et al., 2015; Nguyen et al., 2015). The transcriptional changes of these genes of TCSs in Δ*rsrS* and Δ*rsrR* implied that cross-talk potentially occurs between RsrS-RsrR and other TCSs. Therefore, we propose that the absence of RsrS or RsrR in *A. caldus* might result in the remodulation of the signal transduction pathways and changes in the transcriptional regulatory mechanisms, and certain sulfur-oxidizing pathways may be adjusted to complete the sulfur metabolism. This may be a reasonable explanation for the differences in growth between the mutants and the wild type in S^0^-medium and the ability of Δ*rsrS* and Δ*rsrR* mutants to grow in K_2_S_4_O_6_-medium. In addition, sequences homologous to the 19-bp IRS were not found at any other loci across the whole genomes of three *A. caldus* strains MTH-04, SM-1 and ATCC51756 by using bioinformatics tools (BBS, MP3 and Fimo; Grant et al., 2011; Ma et al., 2013), implying that RsrS-RsrR was specific for regulation of the S_4_I pathway. The discovery of this sequence indicates an important role of the signaling and regulatory systems in efficient metabolism of various RISCs in *A. caldus*. Further, exploration of the two-component and other regulatory systems would provide novel insights to better understand the sulfur metabolism and regulation network in *A. caldus*.

## Conclusion

In this study, we developed a reliable markerless gene knockout method for *A. caldus* and constructed RsrS-RsrR two-component system mutants. We illustrated the regulatory role of RsrS-RsrR on the S_4_I pathway and proposed a tetrathionate-dependent transcriptional regulation model for the two-component system in the S_4_I pathway. Our markerless gene knockout system has many potential applications both in investigations of molecular mechanisms as well as genetic engineering. The elucidation of the mechanism of regulation of the S_4_I pathway by the RsrS-RsrR system helps improve our understanding of molecular mechanisms in the regulation of sulfur metabolism network in *A. caldus*.

## Author contributions

ZW, JQiL, and LC designed, conducted and composed the paper. ZW, YL, CZ, and YW conducted the experiments. BL and RW performed bioinformatics analysis. JQuL, XP, XL, BL, and RW analyzed the data and revised the paper.

## Funding

This work was supported by grants from the Natural Science Foundation (Grant No. 31370138, 31570036), the National Basic Research Program (2010CB630902), the Natural Science Foundation (Grant No. 31400093, 31370084, 30800011), the China Postdoctoral Science Foundation (Grant No. 2015M580585) and the State Key Laboratory of Microbial Technology Foundation (M2015-03), People's Republic of China.

### Conflict of interest statement

The authors declare that the research was conducted in the absence of any commercial or financial relationships that could be construed as a potential conflict of interest.

## Supplementary material

Figure S1 **Multiple alignment of the combined DoxD and DoxA amino acid sequences with homologs**. Conserved residues are shown with black shadow. Accession numbers (GenBank): *Acidithiobacillus caldus* SM-1, DoxD (fused DoxDA), AEK58244; *Acidithiobacillus caldus* MTH-04, fused DoxDA, OAN03452; *Acidithiobacillus caldus* ATCC 51756, DoxD (fused DoxDA), ABP38224; *Acidithiobacillus thiooxidans*, fused DoxDA, WP_024894934; *Acidithiobacillus ferrooxidans*, fused DoxDA, CDQ09967; *Sulfolobus tokodaii*, DoxD and DoxA, NP_377837 and NP_377838; *Sulfolobus solfataricus*, DoxD and DoxA, NP_343149 and NP_343148; *Acidianus ambivalens*, DoxD and DoxA, CAA70827 and CAA70828; *Acidianus ambivalens*, DoxD2 and DoxA2, CAC86936 and CAC86935; *Bacteroides thetaiotaomicron*, fused DoxDA, NP_809428.

Figure S2 **Amino acid sequence and protein structure analysis of RsrR and RsrS**. Every domain is shown in the figure. **(A)** Amino acid sequence alignment between RsrR and OmpR; **(B)** Amino acid sequence alignment between RsrS and EnvZ; **(C)** Protein structure fitting of RsrR and OmpR (blue for RsrR, red for OmpR); **(D)** Protein structure fitting of RsrS and EnvZ (gray for RsrS, red for EnvZ).

Table S1 **Primers used for constructing Δ*rsrR* and Δ*rsrS***.

Table S2 **Primers used for RT-qPCR**.

Table S3 **Primers used for EMSA assays**.

Table S4 **Primers used for constructing IRS-probe vectors**.

Supplementary Text S1 **The original data for the relative transcription levels of genes involved in sulfur metabolism and signaling systems during the S0-cultivating process**.
