# Supplementary figures and images for: The Two-Component System RsrS-RsrR Regulates the Tetrathionate Intermediate Pathway for Thiosulfate Oxidation in Acidithiobacillus caldus
Source: Front Microbiol. 2016 Nov 3;7:1755. doi: 10.3389/fmicb.2016.01755 (PMC5093147; doi:10.3389/fmicb.2016.01755)

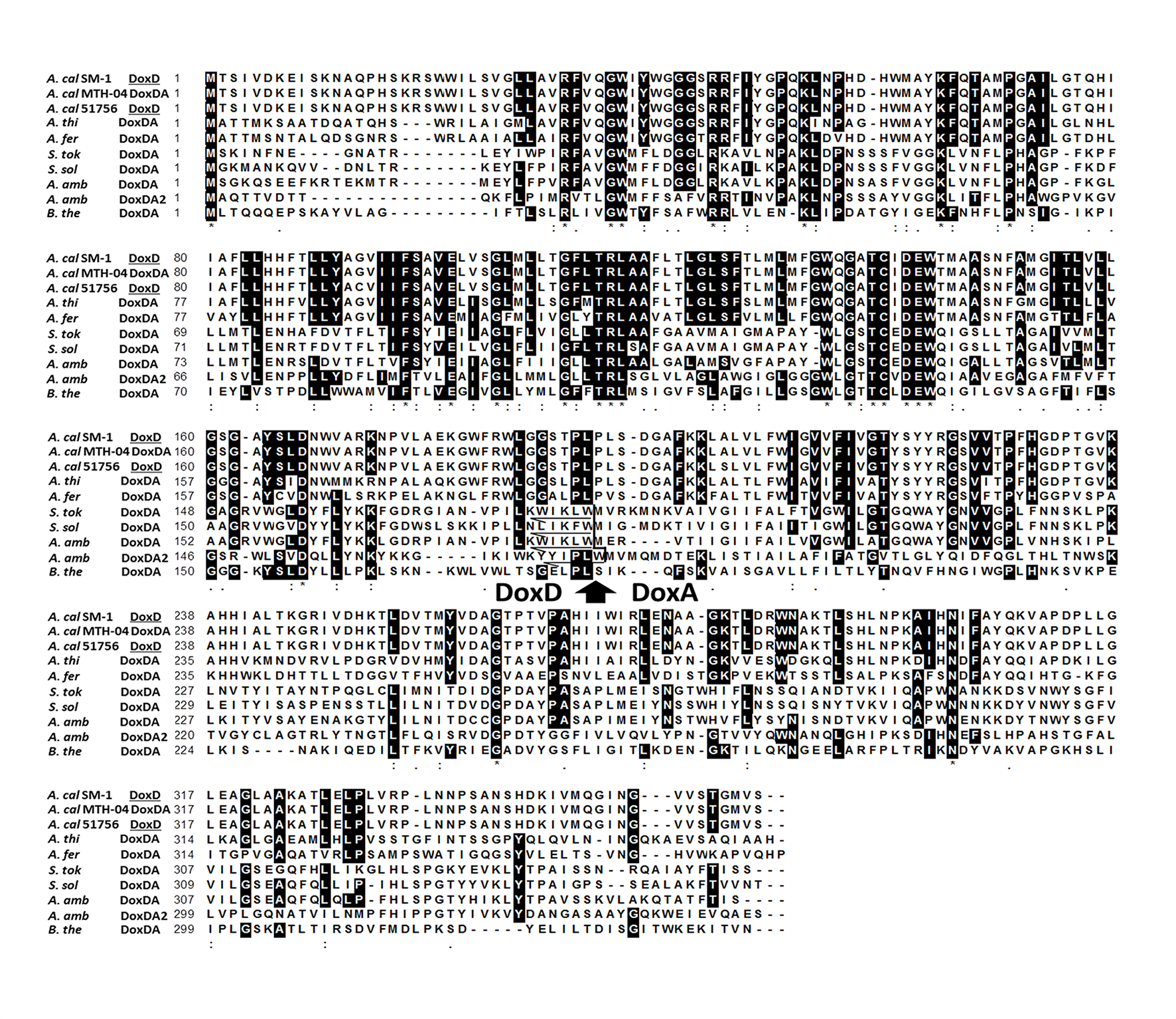

Supplement: Figure S1 — Multiple alignment of the combined DoxD and DoxA amino acid sequences with homologs. Conserved residues are shown with black shadow. Accession numbers (GenBank): Acidithiobacillus caldus SM-1, DoxD (fused DoxDA), AEK58244; Acidithiobacillus caldus MTH-04, fused DoxDA, OAN03452; Acidithiobacillus caldus ATCC 51756, DoxD (fused DoxDA), ABP38224; Acidithiobacillus thiooxidans, fused DoxDA, WP_024894934; Acidithiobacillus ferrooxidans, fused DoxDA, CDQ09967; Sulfolobus tokodaii, DoxD and DoxA, NP_377837 and NP_377838; Sulfolobus solfataricus, DoxD and DoxA, NP_343149 and NP_343148; Acidianus ambivalens, DoxD and DoxA, CAA70827 and CAA70828; Acidianus ambivalens, DoxD2 and DoxA2, CAC86936 and CAC86935; Bacteroides thetaiotaomicron, fused DoxDA, NP_809428. [file Image1.TIF]
