# Supplementary material for: The Two-Component System RsrS-RsrR Regulates the Tetrathionate Intermediate Pathway for Thiosulfate Oxidation in Acidithiobacillus caldus
Source: Front Microbiol. 2016 Nov 3;7:1755. doi: 10.3389/fmicb.2016.01755 (PMC5093147; doi:10.3389/fmicb.2016.01755)
Supplement: Table S1 — Primers used for constructing ΔrsrR and ΔrsrS. [file Table1.DOCX]

**Table S1. Primers used for constructing Δ*rsrR* and Δ*rsrS*.**

| **Primer name** | **Primer Sequence (5'→3')** |
| --- | --- |
| R1F | GACTAGTACGAAAATCTGCGCCTGTCGTA |
| R1R | TGCGTCACCTCTTCTCCACC |
| R2F | TTCCAGCCCAACGGTGGAGAAGAGGTGACGCACGGGGAGAACCGTTAGAGTTATCAC |
| R2R | AAGGAAAAAAGCGGCCGCCCGAATCGCTAATACGTTGTCTAGG |
| R3F | ATATCCAGTTGAGGGTAGAGATTG |
| R3R | CATATCAATTTTCCGGTTCTGGCAT |
| R4F | TCCTGGCTAGAGATCCTGCGGGACT |
| R4R | CACGGTACCTTGAGAGACGAGGAGT |
| R5F | ATCTTCCGCACACATTCTGGTGGT |
| R5R | TCATACATATTTCTGGCTCATCG |
| S1F | ATACGGACTAGTCACAGGAGCATGATCGATGGCA |
| S1R | CCCAAGCTTAGTAGAGCGGTCAACAACA |
| S2F | CCCAAGCTTAGTAACATCGGCACAGAGACAGG |
| S2R | ATATGGGGTACCGTGTTCACTGCACCGGGAATCT |
| S3F | TATTCTGGCGGATGAGATTC |
| S3R | GTAAGGAGCATTAGGCCAGAAAC |
| S4F | TGCGAAGAACAAAGCCCATGAACAG |
| S4R | CATTGTGTGTCGGACTTCCATACGC |
| S5F | GCGAATGACTACCACACTTGAGAAC |
| S5R | GTCGAACAGATTGCGAAATACCCGC |
| RepA sen | CGGGTGCTCTATCGTGTTCCTG |
| RepC ant | CTTCCAAAGGCGCTCGATGC |

* Restriction sites were indicated with underline.
