# Supplementary material for: The Two-Component System RsrS-RsrR Regulates the Tetrathionate Intermediate Pathway for Thiosulfate Oxidation in Acidithiobacillus caldus
Source: Front Microbiol. 2016 Nov 3;7:1755. doi: 10.3389/fmicb.2016.01755 (PMC5093147; doi:10.3389/fmicb.2016.01755)
Supplement: Table S2 — Primers used for RT-qPCR. [file Table2.DOCX]

**Table S2. Primers used for RT-qPCR.**

| **Gene** | **Annotation in genome** |  | **Primer Sequence (5'→3')** |
| --- | --- | --- | --- |
| ***A.caldus*** | |  |  |
| *soxX*-I | A5904_2486 | F | TTCCCCTACGCCAATATGCC |
|  |  | R | CAGAGGTAATCCGCCACCTG |
| *soxY*-I | A5904_2487 | F | CACCCACCATTGCCGAAAAC |
|  |  | R | GTTGTCGGTCTTGGCCATCT |
| *soxZ*-I | A5904_2488 | F | GCCCACTTCATTCAGACCGT |
|  |  | R | TTCAGTGTGCCGCTTTTCTC |
| *soxA*-I | A5904_2489 | F | ATCCTGCCACATGGCTTACG |
|  |  | R | GTACTTTCCAGGGGTTGGGG |
| *soxB*-I | A5904_2491 | F | CGTTTCCGCCACGAAGAAAT |
|  |  | R | CCGGCAGATCGAGCTTTTTG |
| *soxY-*II | A5904_2520 | F | TGGCGAGCGCATTTTTCTTT |
|  |  | R | AACTCACCCTTGTTCGTCCG |
| *soxZ-*II | A5904_2521 | F | AAGCAAGGCAAGCTCATTCC |
|  |  | R | GCGCATCTTGAAGGCAAGG |
| *soxB-*II | A5904_2522 | F | CGTATCACAGACCTGCGTGT |
|  |  | R | CCGGATTATCTCGGTGGTCG |
| *soxX-*II | A5904_2525 | F | GAAGCTGGTGCAGTTTATTTACGA |
|  |  | R | GCCAATCTGGTGATCCGTCA |
| *soxA-*II | A5904_2526 | F | CTCTCCACTTTTGGGGCAGA |
|  |  | R | ATCCGCGCTTTGCAGTTTTT |
| *tetH* | A5904_1013 | F | CAACGGGGCCCGATCTATAC |
|  |  | R | GTTGACCCAATCCCACGAGT |
| *doxDA* | A5904_1014 | F | AGGTTGCTCCGGATCCATTG |
|  |  | R | TTTGTCATGCGAATTGGCCG |
| *sdo* | A5904_0790 | F | TTCACCTTGCCCGAAGAGAC |
|  |  | R | ACCGCTACGTGGATGTGTTT |
| *hdrC* | A5904_1042 | F | GGGTTTCTTCAAGCGTACCG |
|  |  | R | TTGGGCCGAAACAGCGTATT |
| *hdrB* | A5904_1043 | F | GCCGAAGTGGAATTTGGCAT |
|  |  | R | ATCCATGGAATCGCTGACGTT |
| *dsrE* | A5904_2473 | F | TCAGATGACGGTGGACCTCT |
|  |  | R | TGAAAAGGGTGATGTCCGCT |
| *tusA* | A5904_2474 | F | GTGGTCTCAACTGCCCCTTG |
|  |  | R | GCTTCGAAATCCTTCACCGC |
| *rhd-*1 | A5904_0894 | F | CAGCCGGACGAATTTCTGCT |
|  |  | R | CAACCCTCTATCTTGCCTCGT |
| *rhd-*2 | A5904_2860 | F | GACCCATCGTCCGTAAGCTC |
|  |  | R | GATGACGTGATGCTCCTGGT |
| *rhd-*3 | A5904_2475 | F | CCGGGCAGAATTTTCACCTC |
|  |  | R | GGCCTCCGCTCAGACAATAG |
| *rhd-*4 | A5904_1407 | F | GACGGTACAGGGTTGGTTCG |
|  |  | R | TGGCATCGCAGGTAGTTGG |
| *sqr-*1 | A5904_1436 | F | TACCTCAACGAGACCCAAGACA |
|  |  | R | TCGGTCACCACAAATCCCTC |
| *sqr-*2 | A5904_2678 | F | GCGAGACGGTCAAGGAGTTC |
|  |  | R | ACTTCTGGCTCCGCTGATTC |
| *sdo-*1 | A5904_0421 | F | CTTGTCCTACTTCTACGGGTGC |
|  |  | R | GCGTGAATGTGGGTGTCG |
| *sdo-*2 | A5904_0790 | F | TTCACCTTGCCCGAAGAGAC |
|  |  | R | ACCGCTACGTGGATGTGTTT |
| *rsrR* |  | F | ATCGCATACTTGGGTTGGA |
|  |  | R | CCTGTTCATGGGCTTTGTTC |
| *ompR* | A5904_2590 | F | AGGATTTCAGCGTCTGTGGC |
|  |  | R | TTCGGGCTCGAAGGGTTT |
| *phoB* | A5904_0374 | F | GAAAGTGGAGTTGCCTGGTGT |
|  |  | R | AAGGGCTTGGACAGATAGTCG |
| *cheY* | A5904_1450 | F | GCCCTTCGATCTCGTCGTT |
|  |  | R | TTGGCCGCTTCCAGTATGTT |
| *soxR* | A5904_2485 | F | GAGAGCCCTGCACGACGC |
|  |  | R | GCAGCCATCTCCAGTTTACCG |
| *rr* | A5904_0219 | F | CGAGGAACTGCCCTTGGA |
|  |  | R | CGCTGCTTGGGTCACATT |
| *rr* | A5904_0936 | F | GATGGCTCAGGTGGAACGC |
|  |  | R | GCTGGCACCGTGAATGAAGA |
| *rr* | A5904_1207 | F | GGTGGTGATGGACTGGATGTT |
|  |  | R | CTCACGGGCGTTGAAAGG |
| *rr* | A5904_1342 | F | GTCCGCGAACTGGAGAATGC |
|  |  | R | CAAAGGCGGGAAGGCACT |
| *rr* | A5904_1480 | F | GAGCCCTTTGTTGCGGTGAACTGT |
|  |  | R | GGCTTGCTCAAACTTTCCCGGACTG |
| *sr* | A5904_0420 | F | CAACCTTTCCCAGCACCTGA |
|  |  | R | CACCACCGTCGCTAATGC |
| *sr* | A5904_0789 | F | CGGGCGGAACCACCATCTA |
|  |  | R | AGTCCCTCAAAGCCGAGCAG |
| *sr* | A5904_1113 | F | TGCGCCTCCTTGAAACCG |
|  |  | R | GGCAGGACGATGGGAAACA |
| *sr* | A5904_2677 | F | GGAGGTGTTTGCCGAGAATG |
|  |  | R | GGGTTGTAGCGGGAACGAA |
| *rsrS* | A5904_1012 | F | TACCGCCGAATGATAAAC |
|  |  | R | TGGAGAAATACGACGATAAGA |
| *envZ* | A5904_2589 | F | CCTTGGCACCTTCCTCATCG |
|  |  | R | GCACCATTTCCCGCTCTTTC |
| *phoR* | A5904_0373 | F | CGCTGCGACTGGCGTTGGAGGT |
|  |  | R | TCCGTCGCCGTCTCACTCCAAGCTA |
| *cheA* | A5904_1448 | F | CAAACCGCCCACGCCAGCGAA |
|  |  | R | GCCGTCGCCATAGAGCTTATCTA |
| *soxS* | A5904_2484 | F | GGCTGCAGGGAAACCGACATAAGAT |
|  |  | R | ACCCAGCAAACCCAGTAGAGCCGTA |
| *kdpD* | A5904_1340 | F | GGGCCGCGTCTTCGAGCGCTTCTTT |
|  |  | R | GATCTCACCGTGCTGGGCAAGT |
| *fleS* | A5904_1479 | F | AGGGGCTGTCCTCGTTGTCGACGAT |
|  |  | R | TGCTGGAGCAGCCATTCCTCAC |
| *hk* | A5904_0218 | F | CAGCCTCAGCGAGCATCGTCGTGGA |
|  |  | R | ATGCGAGAGTGACCAGGACGAA |
| *hk* | A5904_0934 | F | AGAGGACCTGGGTCATGAACTGGAT |
|  |  | R | GTCCAGACGCACATCCTGCTGCAT |
| *gusA* |  | F | TCAGCGTTGGTGGGAAAG |
|  |  | R | ACGGCGTGACATCGGCTTC |
| *gapdh* | A5904_2603 | F | ACGTCTCCATCGTCGATCTCA |
|  |  | R | AGGGCTTGTCGTTGTAGGCA |
| ***E.coli*** | |  |  |
| *gusA* |  | F | TCAGCGTTGGTGGGAAAG |
|  |  | R | ACGGCGTGACATCGGCTTC |
| *gapdh* |  | F | TGGTCTCAAGCCCAAAGG |
|  |  | R | CGCAGCGTCAAGCGGAAT |
