# Supplementary material for: The Two-Component System RsrS-RsrR Regulates the Tetrathionate Intermediate Pathway for Thiosulfate Oxidation in Acidithiobacillus caldus
Source: Front Microbiol. 2016 Nov 3;7:1755. doi: 10.3389/fmicb.2016.01755 (PMC5093147; doi:10.3389/fmicb.2016.01755)
Supplement: Table S3 — Primers used for EMSA assays. [file Table3.DOCX]

**Table S3.** **Primers used for EMSA assays.**

| **Primer name** | **Primer Sequence (5'→3')** |
| --- | --- |
| rsrR-F | CGGGATCCAAGAGGTGACGCATG |
| rsrR-R | CCCAAGCTTGAGCCACCTAAGC |
| G360-F | CGCAATATTCTGCGGGCGGTC |
| G360-R | TACGGTAAGGTCCACCGGAT |
| T360-F | AGCGCCGATTGTGTACAGAATGAAC |
| T148-F | AATTGTAACACCTGTTACACCT |
| T90-F | ATTGTCTCCTATGGGCCCCGA |
| T360/148/90-R | GATATATAATCTCCGAATCGCTAAT |
| G360+58IRS-R1 | GTTGTAACAGGTGTAACAGGTGTTACAATTTACGGTAAGGTCCACCGGATCCT |
| G360+58IRS-R2 | TGCATATAAGTTTTAGCTTATGTAACAAGTTGTAACAGGTGTAACAGGTG |
| T360Δ19-R | ACAATTTACGTGCGTTGAATTC |
| T360Δ19-F1 | GAATTCAACGCACGTAAATTGTACAACTTGTTACATAAGCTAA |

* Restriction sites were indicated with underline.
