# Supplementary material for: The Two-Component System RsrS-RsrR Regulates the Tetrathionate Intermediate Pathway for Thiosulfate Oxidation in Acidithiobacillus caldus
Source: Front Microbiol. 2016 Nov 3;7:1755. doi: 10.3389/fmicb.2016.01755 (PMC5093147; doi:10.3389/fmicb.2016.01755)
Supplement: Table S4 — Primers used for constructing IRS-probe vectors. [file Table4.DOCX]

**Table S4. Primers used for constructing IRS-probe vectors.**

| **Primer name** | **Primer Sequence (5'→3')** |
| --- | --- |
| PtetH360-sen | ATACCCAAGCTTCTCGAGAGCGCCGATTGTGTACAGAATGAACA |
| PtetH148-sen | ATACCCAAGCTTGAATTCAATTGTAACACCTGTTACACCTGTTA |
| PtetH90-sen | ATACCCAAGCTTGAATTCATTGTCTCCTATGGGCCCCGAGTATA |
| PtetH-ant | GATATATAATCTCCGAATCGCTAAT |
| gusA-sen | ATTAGCGATTCGGAGATTATATATCATGTTACGTCCTGTAGAAACCCCAA |
| gusA-ant | TATCGGGGTACCACTAGTTCATTGTTTGCCTCCCTGCTGCGGT |

* Restriction sites were indicated with underline.
