# Supplementary material for: The Two-Component System RsrS-RsrR Regulates the Tetrathionate Intermediate Pathway for Thiosulfate Oxidation in Acidithiobacillus caldus
Source: Front Microbiol. 2016 Nov 3;7:1755. doi: 10.3389/fmicb.2016.01755 (PMC5093147; doi:10.3389/fmicb.2016.01755)
Supplement: Figure S2 — Amino acid sequence and protein structure analysis of RsrR and RsrS. Every domain is shown in the figure. (A) Amino acid sequence alignment between RsrR and OmpR; (B) Amino acid sequence alignment between RsrS and EnvZ; (C) Protein structure fitting of RsrR and OmpR (blue for RsrR, red for OmpR); (D) Protein structure fitting of RsrS and EnvZ (gray for RsrS, red for EnvZ). [file Image2.pdf]

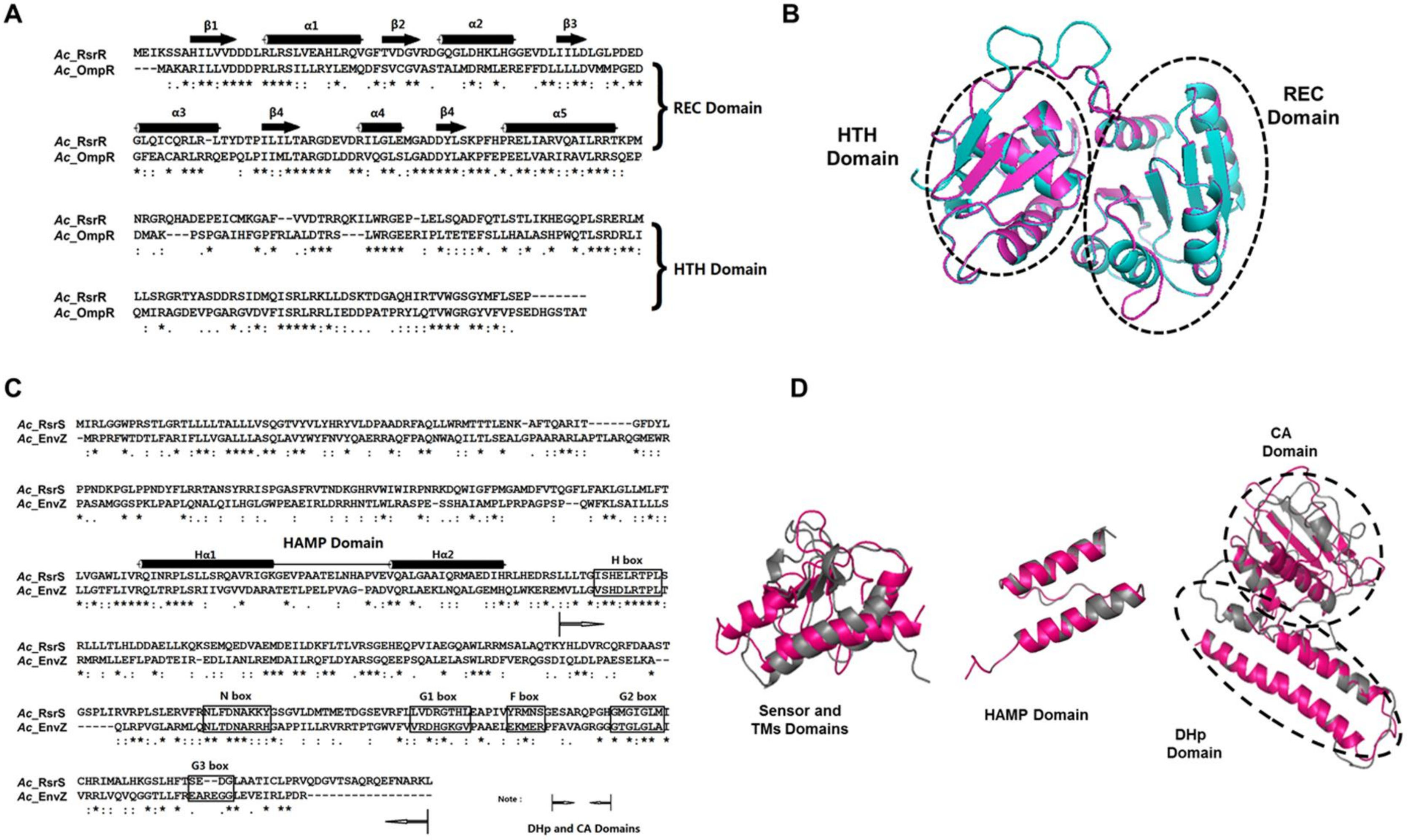

Fig. S2. Amino acid sequence and protein structure analysis of RsrR and RsrS. Every domain is shown in the figure. (**A**) Amino acid sequence alignment between RsrR and OmpR; (**B**) Amino acid sequence alignment between RsrS and EnvZ; (**C**) Protein structure fitting of RsrR and OmpR (blue for RsrR, red for OmpR); (**D**) Protein structure fitting of RsrS and EnvZ (grey for RsrS, red for EnvZ).
